# *NH787* EMS mutant of rice variety Nagina22 exhibits higher phosphate use efficiency

**DOI:** 10.1038/s41598-021-88419-w

**Published:** 2021-04-28

**Authors:** Yugandhar Poli, Veronica Nallamothu, Ai Hao, Muddapuram Deeksha Goud, Xiaowen Wang, Subrahmanyam Desiraju, Satendra K. Mangrauthia, Ajay Jain

**Affiliations:** 1 ICAR-Indian Institute of Rice Research, Hyderabad, 500030 India; 2 State Key Laboratory of Crop Genetics and Germplasm Enhancement, Key Laboratory of Plant Nutrition and Fertilization in Low-Middle Reaches of the Yangtze River, Ministry of Agriculture, Nanjing Agricultural University, Nanjing, 210095 China; 3 Amity Institute of Biotechnology, Amity University Rajasthan, Jaipur, India

**Keywords:** Biochemistry, Biotechnology, Physiology, Plant sciences

## Abstract

Rice (*Oryza sativa* L.), a major dietary source, is often cultivated in soils poor in available inorganic orthophosphate (Pi), which is a key nutrient for growth and development. Poor soils are amended by phosphorus (P) fertilizer, which is derived from the non-renewable rock phosphate reserves. Therefore, there is a need for developing rice varieties with high productivity under low P conditions. At the ICAR-IIRR, ethyl methanesulfonate (EMS) mutagenized rice genotype Nagina22 (N22) were screened for high grain yield in Pi-deprived soil, which led to the identification of ~ 10 gain-of-function mutants including *NH787*. Here, detailed comparative morphophysiological, biochemical, and molecular analyses of N22 and *NH787* were carried out in hydroponics and potting soil under different Pi regimes. Under Pi-deprived condition, compared with N22, *NH787* exhibited higher root and vegetative biomass, the number of tillers, and grain yield. The augmented agronomic traits of *NH787* were corroborated with significantly higher photosynthetic rate, pollen fertility, stigma receptivity, and the activities of antioxidant enzymes superoxide dismutase (SOD) and catalase (CAT). Further, several genes involved in the maintenance of Pi homeostasis (GPH) were differentially regulated. The study thus revealed a wide-spectrum influence of the mutation in *NH787* that contributed towards its higher Pi use efficiency (PUE).

## Introduction

**Figure 1:**
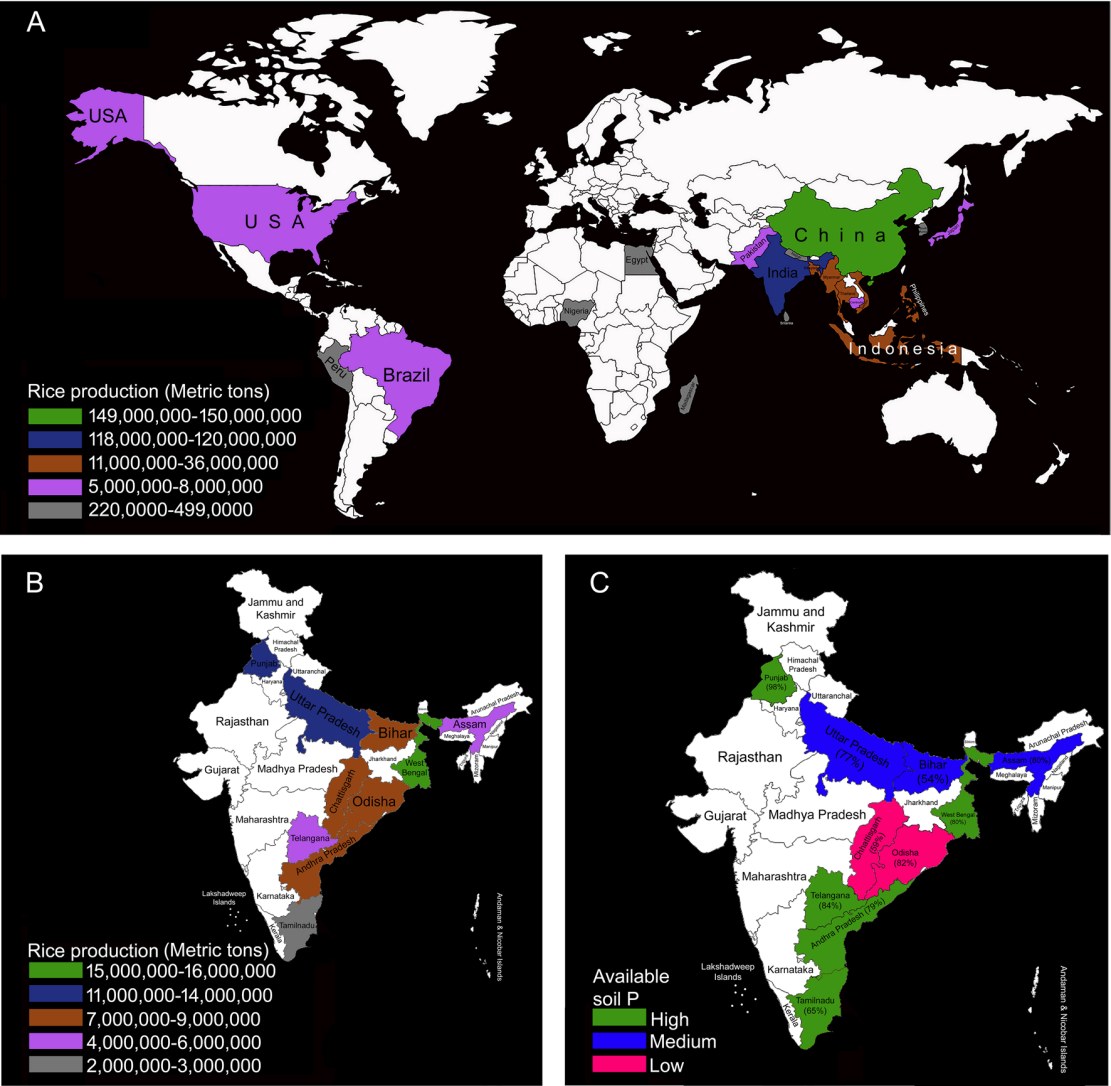
Rice producing (metric tons) (**A**) top 20 countries (www.worldagriculturalproduction.com/crops/rice.aspx) and (**B**) top 10 states in India (www.mapsofindia.com/top-ten/india-crops/rice.html). (**c**) Available soil P content (high, medium, and low) in top 10 states in India producing rice (www.iiss.nic.in/districtmap.html). Photoshop 7.0 version was used to prepare the figure **A**–**C**.

Rice (*Oryza sativa* L.) is the staple food and a major source of dietary energy supply for more than half of the world’s 7.85 billion population (www.worldometers.info/world-population). Rice is consumed ∼ 90% in Asia (www.irri.org/rice-today). China is the world's biggest rice producer among the top 20 rice-producing countries in the world of which 70% are from Asia (Fig. 1A; Table 1). India is the second-largest producer and consumer of rice with ~ 44 million hectares under cultivation and West Bengal, Punjab, and Uttar Pradesh are the top three states in rice production^1^ (Fig. 1B; www.agriexchange.apeda.gov.in). The world population is projected to reach 9.7 billion by 2050 (www.populationpyramid.net/2050) and the percent increase in population in some of the rice-producing Asian countries ranges from 1.80% (Nepal), 15.81% (India) to 34.65% (Pakistan) (Table 1). Therefore, scaling up rice production to achieve sustainable food security for the burgeoning population is warranted.

**Table 1:** Top 20 rice-producing countries, their present (2020), predicted (2050), and per cent increase in population by 2050.

Country	Rice production (metric tons)^a^	Population (in million as of September 2020)^b^	Projected population (in million by 2050)^c^	Per cent increase in population by 2050
China	14,90,00,000	1439.32	1402.40	− 2.63
India	11,80,00,000	1380.00	1639.17	15.81
Bangladesh	3,60,00,000	164.68	192.56	14.48
Indonesia	3,49,00,000	273.52	330.90	17.34
Vietnam	2,75,00,000	97.33	109.60	11.2
Thailand	2,04,00,000	69.79	65.94	− 5.84
Myanmar	1,31,00,000	54.40	62.25	12.61
Philippines	1,10,00,000	109.58	144.48	24.16
Japan	76,50,000	126.47	105.80	− 19.54
Pakistan	75,00,000	220.89	338.01	34.65
Brazil	68,68,000	212.55	228.98	7.18
United States	68,64,000	331.00	379.41	12.76
Cambodia	57,80,000	16.71	21.86	23.56
Nigeria	49,61,000	206.13	401.31	48.64
Egypt	43,00,000	102.33	159.95	36.02
South Korea	37,44,000	51.26	NA	NA
Nepal	36,75,000	29.13	35.32	17.53
Sri Lanka	28,93,000	21.41	21.81	1.83
Madagascar	26,88,000	27.69	54.04	48.76
Peru	22,77,000	32.97	40.37	18.33

*NA* not available.

^a^www.worldagriculturalproduction.com/crops/rice.aspx

^b^www.worldometers.info/world-population.

^c^www.populationpyramid.net/2050.

Phosphorus (P), one of the essential macroelements, is a building block of various organic molecules such as ATP, nucleic acids, and phospholipids, and also plays a key role in energy transfer, signal transduction, metabolic pathways, and thus indispensable for the proper growth and development of plants^2–6^. In the rhizosphere, P is largely available in the form of inorganic orthophosphate (Pi) and its acquisition by the roots and subsequent translocation to various parts of the plants is mediated by a suite of Pi transporters^7–9^. However, rice is often cultivated in a rain-fed system on soils subjected to various abiotic stresses including poor availability and/or fixing of P, which adversely affects yield potential^10,11^. Rice in India is normally produced in soils poor in Pi availability and largely amended by application of P fertilizer^12,13^ (Fig. 1C). P fertilizer is produced from the non-renewable and finite rock phosphate (phosphorite) reserves likely to be exhausted in the next 50–100 years at the current rate of its usage across the globe^14^. Therefore, there is an urgent need to identify or develop rice varieties with higher PUE under low P conditions^15,16^.

Sequencing of the whole rice genome and an efficient transformation system has made it a favored model monocotyledonous plant^17–19^. The arduous task of the post-genomic era has been to systematically evaluate the function of an array of diverse GPH in rice. Loss-of-function mutagenesis (T-DNA and *Tos17*)-mediated reverse genetics has significantly contributed to the functional genomics of rice^20,21^. RNAi-mediated gene-silencing has also been an attractive approach for functional genomics^22^. A programmable CRISPR/Cas9 system emerged as a promising molecular tool for genome editing^23^and Jennifer Doudna and Emmanuelle Charpentier were awarded the 2020 Nobel prize in Chemistry for developing this versatile technology. CRISPR/Cas9 system is now a favored technology for generating transgene-free rice plants^24–26^. A gain-of-function mutagenesis is an alternative approach based on the ectopic overexpression of transgenes under the control of a strong constitutive *CaMV35S* or ubiquitin promoter^27^. Functional characterization of several GPH by reverse and/or forward genetic approach has thus led to the identification of several key positive and negative regulators of sensing and signaling cascades governing the maintenance of Pi homeostasis^3,6,28^ (Table 2). However, plants generated by these forward and reverse genetics approaches are often deemed as a potential transgene and are regulated by stringent country-specific ethical legislations, and often fail to comply with the biosafety regulations^29–31^. Although CRISPR-edited rice was considered to comply with the regulatory approval for commercial applications^24^, recently Court of Justice of the European Union has clubbed them with GM plants^32–34^. One of the classical controversial cases is the Golden rice, which was engineered to produce seeds enriched with β-carotene to mitigate vitamin A deficiency in the millions of poor people^35^ but has been embroiled in polarized debate over its ethicality^36^. On the contrary, mutation breeding by exposure to mutagens such as EMS or irradiation by X-rays is environmentally benign, has good safety records, and is not regulated worldwide^29^. EMS-induced mutagenesis is an attractive strategy for inducing genetic variations in the genome^37,38^ and has facilitated the development of a rich repository of rice mutants that exhibit tolerance to different biotic and/or abiotic stresses^39^. N22 is an upland and short *aus* genotype and is tolerant to heat and drought^40,41^. An initiative was launched by the Department of Biotechnology, Govt. of India, for generating EMS-mutagenized M2 populations (~ 85,000) in the background of N22^41^. At the ICAR-IIRR, efforts are underway for more than a decade to screen N22 EMS mutants that exhibited altered PUE under field condition, which led to the identification of several loss-of-function and gain-of-function mutants^42–47^. Among these mutants, detailed morphophysiological and molecular analyses were carried out for the loss-of-function mutant *NH101*, which revealed several traits that were affected contributing towards its lower PUE compared with N22^47^.

**Table 2:**
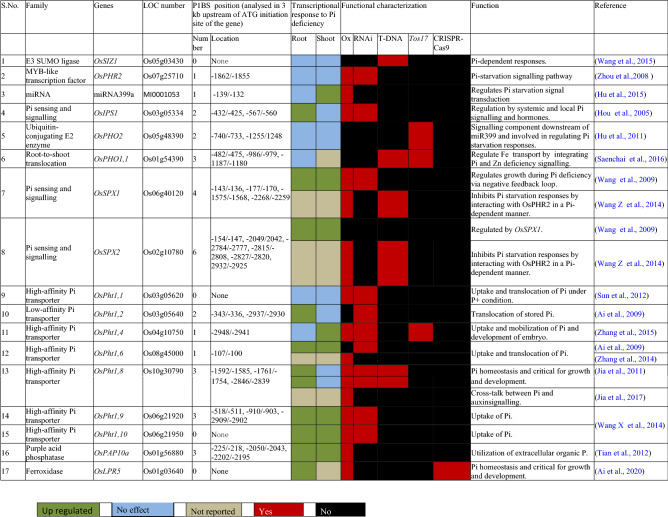
Functionally characterized genes involved in the maintenance of Pi homeostasis in rice.

However, gain-of-function N22 mutant that shows significantly higher PUE has not been characterized as yet. Therefore, in this study, detailed comparative morphophysiological, biochemical, and molecular analyses of N22 and *NH787* mutant were carried out in the hydroponics and potting soil under different Pi regimes. The analysis revealed several traits that contributed towards the higher PUE of the gain-of-function *NH787* mutant.

## Materials and methods

### Plant materials and experimental conditions

Rice (*Oryza sativa* L. ssp *indica*) genotype N22 were mutagenized with EMS and several gain-of-function mutants i.e., *NH363*, *NH514*, *NH686*, *NH719*, *NH776*, and *NH787* were identified, which exhibited high grain yield in Pi-deprived soil under field condition compared with N22^45^. From these gain-of-function mutants, *NH787* was selected for detailed morphophysiological, biochemical, and molecular analyses. About 15 seeds each of N22 and *NH787* were placed equidistant on a Pteri plate lined with germination paper soaked with deionized distilled water and wrapped in aluminum foil and kept for germination in a growth chamber (28–30 °C) for 4 days. N22 and *NH787* seedlings showed significant variation in their radicle length. Therefore, the seedlings were scanned and their radicle was measured by employing the ImageJ program^48^ and only those in the range of 2.0–3.0 cm were selected and transferred to the hydroponic system as described^49^ containing P+ (0.3 mM NaH_2_PO_4_) and P− (0 mM NaH_2_PO_4_) media as described^50^ for 7 days. For the potting soil experiment, N22 and *NH787* were grown initially under normal soil condition for 14 days. Subsequently, the seedlings were transplanted in earthen pots which were filled with 8 kg of normal soil (P+) and low P soil (P−) with the Olsen P values of 24 kg/ha and 1.8 kg/ha for P+ and P− soil, respectively. P+ and P− soils were fertilized as described^47^. All plant experiments were carried out in accordance with the guidelines and permission was obtained to collect the rice seeds.

### Quantitative analysis of the root traits

Seedlings grown in the hydroponic system were removed along with the mesh after 7 days treatment under P+ and P− conditions and placed in an inverted position in a Petri plate containing a pool of water. Under the stereomicroscope, roots were separated at the shoot: hypocotyl junction and transferred to a Petri plate containing 1% (w/v) agar. Adventitious, seminal, and lateral roots were spread gently with a camel hair brush to reveal the root system architecture (RSA). Spread-out roots were then scanned at 1000 dpi using a desktop scanner. Scanned images were then used for documenting the number and length of different root traits by using the ImageJ program^48^.

### Quantitative analysis of agronomic traits

Plants grown in the potting soil (P+ and P−) up to maturity were quantitatively analyzed for the growth performance, biomass and length of root, vegetative biomass, number of tillers, filled spikelets/panicle, unfilled spikelets/panicle, 100-grain weight, and yield as described^47^.

### Quantitative analysis of physiological traits

Plants were grown in the potting soil (P+ and P−) up to 50% flowering and flag leaf was assayed for photosynthetic rate (*P*_N_), stomatal conductance (*g*_s_), intercellular CO_2_ concentration (*C*i), and transpiration rate (*E*) by using portable photosynthesis system LI-6400XT (LI-COR Biosciences, USA) set at 1200 μmol m^–2^ s^–1^ photosynthetically active radiation (PAR) and 387 ± 6 ppm CO_2_ concentration. Coefficient of photochemical quenching (qP), coefficient of non-photochemical quenching (qN), electron transport rate (ETR), and maximum efficiency of PSII photochemistry (Fv/Fm) were quantified by employing portable chlorophyll fluorometer PAM-2100 (Heinz Walz GmbH, Germany). Chlorophyll a, b, and carotenoids were extracted and their concentrations were quantified as described^51, 52^.

### Quantification of soluble Pi

Harvested root and shoot were rinsed thoroughly 4–5 times with deionized distilled water, blotted-dry, frozen in liquid nitrogen, ground to a fine powder, and stored at – 80 °C till further use. Ground tissue (~ 25 mg) was homogenized in 200 μl of 1% (v/v) glacial acetic acid, vortexed, and centrifuged at 10,000 rpm for 10 min to remove the debris. The supernatant was collected for the quantification of soluble Pi by phosphomolybdate colorimetric assay as described^53^.

### Quantification of APase enzyme activity

APase enzyme activity was quantified as described^54^ with minor modifications. Freshly harvested root and shoot tissues (~ 0.1 g) were ground in a chilled citrate buffer (0.1 M, pH 5.2), centrifuged at 12,000 rpm at 4 °C for 15 min, and the supernatant was assayed for APase enzyme activity. The reaction mixture comprised 0.1 ml supernatant, 0.4 ml chilled citrate buffer (0.1 M, pH 5.2), and 0.5 ml p-nitrophenol (pNP) (10 mM, pH 5.2). The reaction mixture was incubated at room temperature for 10 min and the reaction was then terminated by adding 2 ml of Na_2_CO_3_ (0.2 M). The standard curve was prepared with the known concentrations of pNP and APase enzyme activity was computed by estimating the accumulation of pNP at 405 nm.

### Quantification of antioxidant enzyme activities

Freshly harvested root and shoot tissues (~ 0.1 g) were ground in phosphate buffer (0.1 M, pH 7.5) containing EDTA (0.5 mM) and centrifuged at 12,000 rpm at 4 °C for 15 min. The supernatant was collected for assaying the activities of different antioxidant enzymes. Superoxide dismutase (SOD) was assayed as described^55^. The reaction mixture (1.5 ml phosphate buffer [100 mM, pH 7.8], 0.2 ml methionine [200 mM], and 0.1 ml each of the plant extract, Na_2_CO_3_ [1.5 M], EDTA [3.0 mM], NBT [2.25 mM], and riboflavin [60 μM]) was incubated under a fluorescent lamp (15 W) for 15 min. SOD activity was determined by a 50% decrease in the absorbance at 560 nm due to rapid inhibition of O_2_^−^ with NBT. Peroxidase (POD) activity was assayed as described^56^. The reaction mixture comprised 1.0 ml phosphate buffer (100 mM, pH 6.1), 0.5 ml each of guaiacol (96 mM), H_2_O_2_ (12 mM), and 0.1 ml of the enzyme extract. The absorbance was taken at 470 nm at different time intervals (0, 1, 2, and 3 min). Catalase (CAT) was assayed as described^57^. The reaction mixture comprised 1.5 ml phosphate buffer (100 mM, pH 7.0), 0.5 ml H_2_O_2_ (75 mM), and 0.05 ml of the enzyme extract. A temporal disappearance of H_2_O_2_ was recorded at an interval of 30 s for 2 min at 240 nm. Ascorbate peroxidase (APX) activity was assayed as described^58^. The root and shoot tissues were ground in a solution containing 1.5 ml phosphate buffer (100 mM, pH 7.0) containing ascorbic acid (1 mM), and EDTA (0.5 mM). The solution was centrifuged at 12,000 rpm at 4 °C for 20 min and the supernatant was collected for the assay. The reaction mixture comprised 1.5 ml phosphate buffer (100 mM, pH 7.0), 0.1 ml each of EDTA (3.0 mM), H_2_O_2_ (3.0 mM), 0.5 ml ascorbic acid (3 mM), and 0.05 ml of the enzyme extract. The APX activity was measured by monitoring the gradual decrease in the absorbance value at an interval of 30 s for 2 min at 290 nm.

### Quantification of H_2_O_2_ content

H_2_O_2_ content was estimated as described^59^. Freshly harvested root and shoot tissues (~ 0.5 g) were ground in 10 ml of trichloroacetic acid, centrifuged at 12,000 rpm at 4 °C for 15 min, and the supernatant was collected for the assay. The reaction mixture comprised 0.5 ml of phosphate buffer (10 mM, pH 7.0), 2 ml of KI (1 M), and 0.5 ml of the supernatant. The reaction mixture was vortexed for 1 min, incubated in dark for 30 min, and H_2_O_2_ content was quantified at 390 nm.

### Assay for pollen viability

The anthers from the spikelets were collected just before anthesis, crushed in Lugol's (I_2_-KI) solution, and observed under a light stereomicroscope as described^60^. Sterile and fertile pollens were unstained and stained, respectively and their images were captured using a compound microscope (10X).

### Quality traits

Harvested grains were threshed, cleaned, and dried at 45 °C for 3 days to achieve identical moisture content. Grains (~ 25 g) were dehulled using a sheller (Satake Co. Ltd. Japan). The hulling rate was computed as described^61^. Brown rice was milled by employing Pearlest grain polisher (Kett, USA) and the milling rate was calculated as described^61^. The head rice recovery was calculated by weighing polished rice and separating head rice (≥ ¾ length of the brown rice) manually from the broken fractions. Gel consistency (GC) was computed as described^62^. Gelatinization temperature was calculated based on the alkali spread score of the milled rice as described^61^. Amylose content was estimated from the ground rice flour calorimetrically as described^63^. Length, width, and area of grains were measured by using the ImageJ program^48^.

### qRT-PCR analysis

Total RNA (~ 2 μg) was isolated from the ground tissue using Trizol reagent and treated with RNase-free DNase. First-strand cDNA was synthesized by using oligo (dT)-18 primer and Superscript II Reverse Transcriptase (Invitrogen). *OsActin* (LOC_Os03g50885) was used as an internal control. The qRT-PCR analysis was performed in triplicate using SYBR Premix Ex TaqII (TaKaRa) in a StepOnePlus Real-time PCR system (Applied Biosystems). Relative expression levels of the genes were computed by the 2^–ΔΔ*C*^_T_ method of relative quantification^64^. Gene-specific primers are listed in Supplementary Table S1.

### Statistical analysis

Two-way analysis of variance (ANOVA) was performed using open-source software R^65^ with agricolae package. Statistical significance of the parameter means was determined by performing Fisher's LSD test.

### Ethical approval

The authors declare that the experiments comply with the current laws of the country in which they were performed and in compliance with ethical standards.

## Results and discussion

### Selection of the uniformly grown seedlings for treatment under different Pi regimes in a hydroponic system

Easy-to-assemble, element-contamination-free, and the aseptic hydroponic system is suitable for documenting the developmental responses of different root traits of the rice seedlings grown under different Pi regimes^45,47,49^. The seed area of N22 and *NH787* was documented by employing the ImageJ program^48^. There was no significant variation in the seed area of N22 and *NH787* (Fig. S1a). Relatively, the seed area was marginally higher (~ 5%) in N22 EMS mutant *NH101*^47^. This suggested a variable effect of EMS mutagenesis on the seed area of N22 EMS mutants. Seeds (~ 20) of N22 and *NH787* were placed equidistant on a Petri plate lined with a germination paper soaked with deionized distilled water, wrapped in aluminum foil, and maintained in a growth chamber (28–30 °C) for 4 days. Rice seed with radicle length > 0.5 cm was considered germinated^47^. The images of germinated seedlings (~ 200 each of N22 and *NH787*) spread over 10 Petri plates were captured by using a desktop scanner (Fig. S1b). A significant variation was apparent in the radicle length of the germinated seedlings of both N22 and *NH787.* Earlier studies had suggested selecting only those rice seedlings whose radicle length falls within a fairly comparable size range (~ 2 to 3 cm) for subsequent transfer to a hydroponic system under different Pi regime to circumvent any erroneous interpretations^47,49^. In the model plant *Arabidopsis thaliana* also, the selection of uniformly grown seedlings with primary root length in the range of ~ 1.5 to 2.5 cm was recommended to minimize the effect of intrinsic variability on the subsequent treatments under different Pi regimes^66–68^. Therefore, the radicle length of the germinated N22 and *NH787* seedlings was measured by using the ImageJ program^48^ and categorized into different groups based on their radicle length (Fig. S1c). The size distribution pattern of N22 and *NH787* radicle length is represented by the red (≤ 0.50 cm), black (0.51–2.0 cm), green (2.01–3.00 cm), and yellow (3.01–5.50) histograms, which exhibited a typical Gaussian curve and a noticeable variation between the genotypes. The number of seedlings with radicle length in the size range of 2.01–3.00 cm was significantly higher (49.38%) in *NH787* compared with N22 (21.88%). These seedlings were eventually selected for transfer to the hydroponic system containing P+ and P− media and the rest of the seedlings (< 2.01 cm and > 3.00 cm) were discarded (Fig. S1d).

### Responses of ontogenetically distinct root traits under different Pi regimes in a hydroponic system

**Figure 2:**
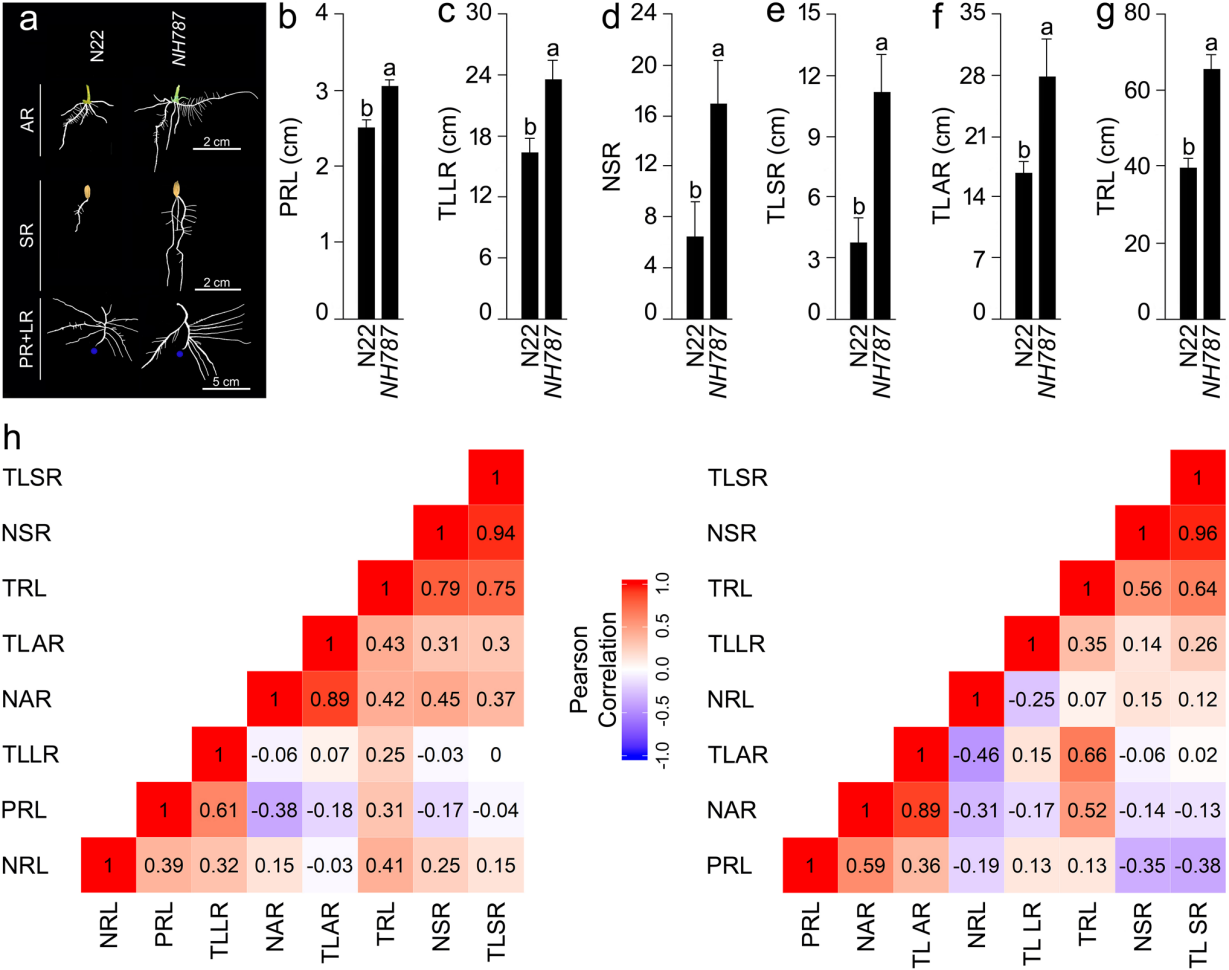
Effects of Pi deficiency on different RSA traits. N22 and *NH787* seedlings (4-day-old) were grown hydroponically under P− condition for 7 day. (**a**) Roots were spread gently and scanned to reveal the architectural details. The primary root tip is indicated by a blue dot. (**b**–**g**) Data presented for (**b**) primary root length (PRL), (**c**) total length of the lateral roots (TLLR), (**d**) number of seminal roots (NSR), (**e**) total length of seminal roots (TLSR), (**f**) total length of adventitious roots (TLAR), and (**g**) total root length (TRL). Values (*n* = 12) are means ± SE and different letters on the histograms indicate that the means differ significantly (*P* < 0.05). (**h**) Correlogram of the RSA traits in Pi-deprived N22 and *NH787.* The scale represents Pearson correlation values with reddish and bluish shades indicate positive and negative correlation, respectively.

The root system of rice comprises ontogenetically distinct embryonically developed primary and seminal roots that play a key role during the seedling stage and post-embryonically developed adventitious roots constitute the bulk of the functional root system in a mature plant^69,70^. N22 and *NH787* seedlings (4-day-old) with radicle length in the size range of 2.01–3.00 cm were transferred to the hydroponic system containing P+ and P− media and grown for 7 d. After the treatment, roots of N22 and *NH787* were separated at the shoot: hypocotyl junction and spread gently to reveal the architectural details of the embryonically and post-embryonically developed traits under P+ and P− conditions. Images of the spread-out roots were captured by using a desktop scanner and the ImageJ program^48^ was then used for quantitative documentation of the effects of P+ (Fig. S2B) and P− (Fig. 2b–g) treatments on different root traits. There was a significant reduction (31.23%) in the primary root length (PRL) of N22 under P− condition (data not shown) and the result was consistent with earlier studies on N22^47,49, 71^. On the contrary, PRL of *NH787* was comparable under P+ and P− conditions (data not shown). Although PRL of N22 and *NH787* was comparable under P+ condition, it was significantly higher (21.56%) in the latter compared with the former under P− condition (Fig. 2a,b). The number of lateral roots (NLR) was significantly reduced (47.31%) in *NH787* compared with *N22* under P+ condition (Fig. S2A,B) but was comparable under P− condition (data not shown). Pi deficiency triggered a significant reduction (72.01%) in the total length of the lateral roots (TLLR) on primary, seminal, and adventitious roots of N22 (data not shown) and agreed with earlier studies on N22^47,49^. Relatively, Pi deficiency-mediated reduction of TLLR in *NH787* was 48.64%, which was significantly lower compared with N22 (data not shown). This suggested that the effect of Pi deficiency on TLLR was more aggravated on N22 than *NH787.* Although TLLR of N22 and *NH787* was comparable under P+ condition (Fig. S2A), it was significantly higher (43.42%) in the latter compared with the former under P− condition (Fig. 2a,c). In rice, elongation of the seminal root plays a key role in the acquisition of nutrients such as Pi and nitrogen (N)^72^. Therefore, the effect of Pi deficiency was investigated on the number of seminal roots (NSR) and the total length of seminal roots (TLSR) of N22 and *NH787.* The effect of Pi deficiency was evident on the developmental response of the seminal roots of N22, which revealed significant reductions by 90.03% and 84.68% in their NSR and TLSR, respectively compared with P+ condition (data not shown) and was congruent with studies on N22^47,49^. The effects of Pi deficiency on both NSR and TLSR of *NH787* were relatively less aggravated and resulted in reductions by 69.88% and 55.99%, respectively (data not shown). NSR and TLSR of N22 and *NH787* were comparable under P+ condition (Fig. S2a). However, under P− condition the NSR and TLSR of *NH787* were 2.6-fold and 2.9-fold higher, respectively compared with N22 (Fig. 2a,d,e). Pi deficiency has also been shown to exert an attenuating influence on the seminal root length of rice varieties *O. rufipogon* (IRGC 105491) and Curinga^72^. The total length of adventitious roots (TLAR) increased significantly (25.52%) in N22 during Pi deficiency (data not shown) and the result was in agreement with earlier studies on N22^47,49^. Relatively, the increase in TLAR was only 12.29% in Pi-deprived *NH787* (data not shown). TLAR of N22 and *NH78*7 was comparable under P+ condition (Fig. 2a) but was significantly higher (65.53%) in the latter compared with the former under P− condition (Fig. 2a,f). Finally, the total root length (TRL) was computed by summation of PRL, TLLR, TLSR, and TLAR. Pi deficiency exerted a significant (63.38%) attenuating effect on the TRL of N22 (data not shown). Earlier studies also reported the inhibitory effect of Pi deficiency on TRL of rice varieties N22^47,49^ and IR64 (transgenics [null] and NILs with [+] or without [−] *Pup1*)^10^. Comparatively, the effect of Pi deprivation was less aggravated on TRL of *NH787* and exhibited a 36.74% reduction compared with P+ condition (data not shown). Although TRL of N22 and *NH787* was comparable under P+ condition (Fig. S2A), it was significantly higher (65.68%) in the latter compared with the former under P− condition (Fig. 2a–g). Together, the detailed analyses of different root traits revealed that the effects of Pi deficiency were more aggravated on N22 than *NH787.* Further*,* the Pearson correlation was presented as a correlogram to determine the relationship across the developmental responses of ontogenetically distinct root traits of N22 and *NH787* under different Pi regimes (Fig. S2C, Fig. 2h). Under P+ condition, TLLR was positively and significantly correlated with TRL and NSR in N22, whereas a significant positive correlation was observed between NLR, TLLR, and TRL and NAR and TLAR in *NH787* (Fig. S2C). Under P− condition, NSR and TLSR in N22, and TLSR, NAR, TLAR, and TRL in *NH787* exhibited a significant positive correlation (Fig. 2h). The analysis revealed that NSR, TLSR, and TLLR were positively and significantly correlated with TRL in both Pi-deprived N22 and *NH787.* Pearson correlation has also been used in earlier studies on various morpho-biochemical traits at various developmental stages of N22 and its EMS mutants under different Pi regimes^43,44,47^.

### Effects of different Pi regimes on various morpho-agronomic traits of N22 and NH787 grown to maturity in potting soil

**Figure 3:**
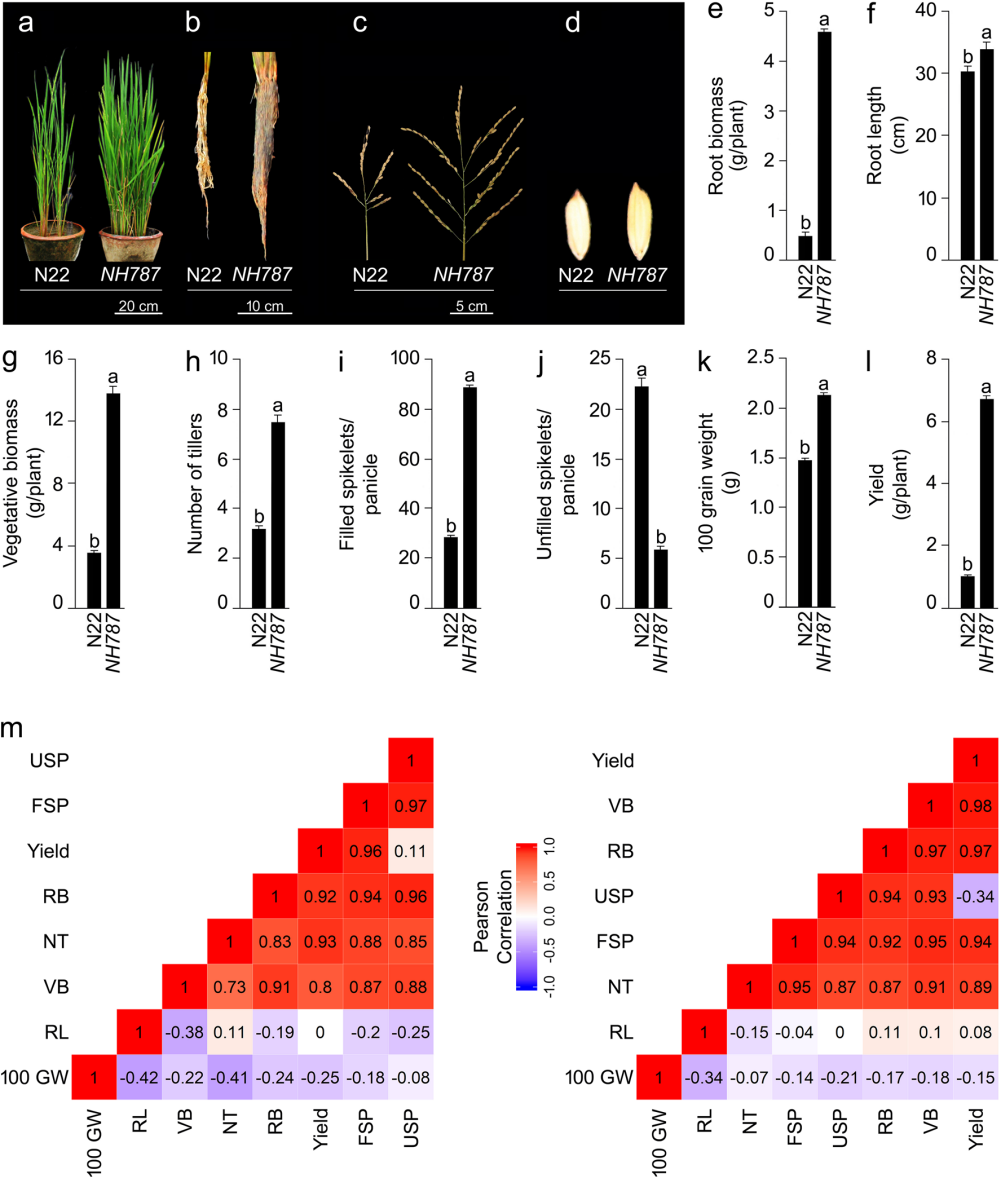
Effects of Pi deficiency on the growth performance and agronomic traits. N22 and *NH787* seedlings (15-day-old) were grown in a low Pi (P−) potting soil up to maturity. (**a**–**d**) Growth performance (**a**) and phenotype of the root (**b**), panicle (**c**), and seed (**d**) of Pi-deprived N22 and *NH787.* The photographs (**a**–**d**) are representatives of 12 independent biological replicates. (**e**–**l**) Data presented for (**e**) root biomass, (**f**) root length, (**g**) vegetative biomass, (**h**) number of tillers, (**i**) filled spikelets/panicle, (**j**) unfilled spikelets/panicle, (**k**) 100-grain weight, and (**l**) yield. Values (*n* = 12) are means ± SE and different letters on the histograms indicate that the means differ significantly (*P* < 0.05). Correlogram of agronomic traits i.e., filled spikelets/panicle (FSP), number of tillers (NT), root biomass (RB), root length (RL), vegetative biomass (VB), unfilled spikelets/panicle (USP), and 100-Grain weight (100 GW) in Pi-deprived N22 and *NH787.* The scale represents Pearson correlation values with reddish and bluish shades indicate positive and negative correlation, respectively.

Growth performance and morpho-agronomic traits of N22 and *NH787* plants grown to maturity (50% flowering) in Pi-replete (P+) and low Pi (P−) potting soil were determined (Fig. S3, Fig. 3). Pi deficiency exerted inhibitory effects on various morpho-agronomic traits of both N22 and *NH787*, which resulted in stunted phenotype, and significant reductions in the root biomass (N22 [88.37%], *NH787* [24.59%]), vegetative biomass (N22 [73.48%], *NH787* [12.03%]), filled spikelets/panicle (N22 [50.18%], *NH787* [17.53%]), 100-grain weight (N22 [25.00%], *NH787* [8.69%]), and yield (N22 [83.08%], *NH787* [23.59%]) (data not shown). Whereas, during Pi deficiency, the unfilled spikelets/panicle was significantly higher in N22 (52.74%) but was comparable in *NH787* with P+ condition (data not shown). It was evident from the analysis that the effects of Pi deprivation were relatively more aggravated in N22 than *NH787*. Earlier studies also reported the inhibitory effects of Pi deficiency on various morpho-agronomic traits of N22 and its EMS mutants^42–44,46,47,71^. Under P+ condition, there was no apparent difference in the phenotype of N22 and *NH787* (Fig. S3A). However, the phenotype of the root, panicles, and grain was more robust in *NH787* compared with N22 (Fig. S3B-D). This was reflected in significantly higher root biomass (41.86%), vegetative biomass (19.70%), filled spikelets/panicle (89.79%), 100-grain weight (15.00%), and yield (36.96%) of *NH787* compared with N22 (Fig. S3E–G,I,J). On the contrary, unfilled spikelets/panicle was significantly higher (61.64%) in N22 compared with *NH787* (Fig. S3H). However, under P+ condition the root length and number of tillers in N22 and *NH787* were comparable (data not shown). Further, Pearson analysis revealed a positive and significant correlation of yield with root and vegetative biomass, number of tillers, and filled spikelets/panicle in both N22 and *NH787* under P+ condition (Fig. S3K). Under P− condition, the phenotype of the plant, root, panicles, and grain were more robust in *NH787* than N22 (Fig. 3a–d). The phenotypic observation was substantiated with significantly higher root biomass (9.2 folds), root length (12.21%), vegetative biomass (3.9 folds), number of tillers (2.37 folds), filled spikelets/panicle (3.15 folds), 100-grain weight (40.00%), and yield (6.42 folds) of *NH787* compared with N22 (Fig. 3e–i,k,i). However, unfilled spikelets/panicle was significantly higher (73.99%) in N22 than *NH787* (Fig. 3j). Similar to P+ condition, under P− condition also Pearson analysis showed a positive and significant correlation of yield with root and vegetative biomass, number of tillers, and filled spikelets/panicle in both N22 and *NH787* (Fig. 3h).

### Photosynthetic and chlorophyll fluorescence traits of N22 and *NH787* grown to maturity in potting soil

**Figure 4:**
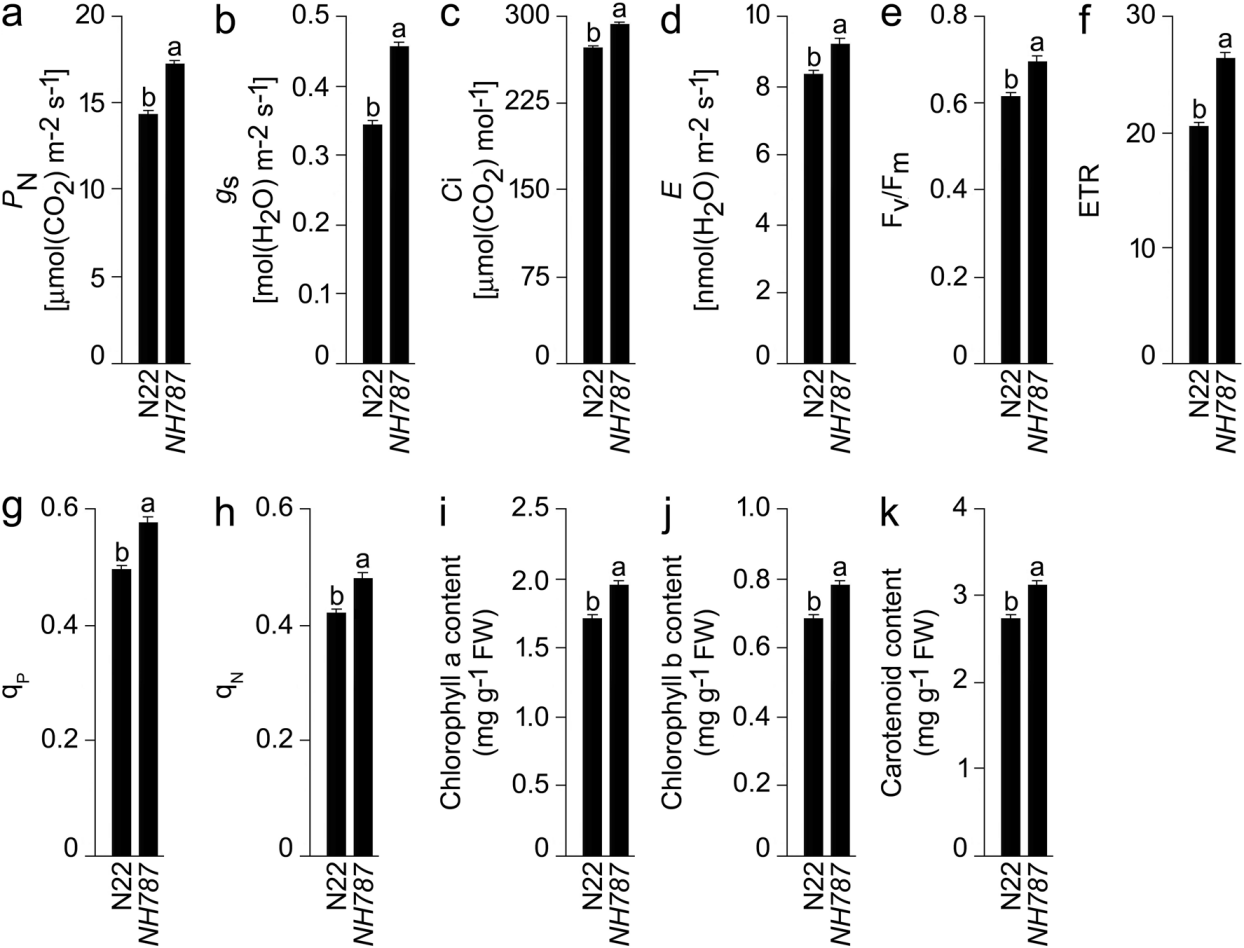
Effects of Pi deficiency on the photosynthetic and fluorescence traits in N22 and *NH787*. (**a**–**k**) Data are presented for (**a**) Photosynthetic rate (*P*_N_), (**b**) Stomatal conductance (*g*_s_), (**c**) Intercellular CO_2_ concentration (*C*i), (**d**) Transpiration rate (*E*), (**e**) Maximum efficiency of PSII photochemistry (Fv/Fm), (**f**) Electron transport rate (ETR), (**g**) Coefficient of photochemical quenching (qP), (**h**) Coefficient of non-photochemical quenching (qN), and contents of (**i**) Chlorophyll a, (**j**) Chlorophyll b, and (**k**) carotenoid. Values (*n* = 12) are means ± SE and different letters on the histograms indicate that the means differ significantly (*P* < 0.05).

Pi deficiency adversely affects photosynthetic and chlorophyll fluorescence traits in rice^73,74^. Therefore, photosynthetic and fluorescence traits were assayed in N22 and *NH787* grown to maturity under different Pi regimes (Fig. S4, Fig. 4). Pi deficiency triggered significant reductions in the photosynthetic rate (*P*_N_) (N22 [21.84%], *NH787* [26.39%]), stomatal conductance (*g*_s_) (N22 [88.24%], *NH787* [42.23%]), transpiration rate (*E*) (N22 [15.31%], *NH787* [27.90%]), maximum efficiency of PSII photochemistry (Fv/Fm) (N22 [15.79%], *NH787* [8.73%]), electron transport rate (ETR) (N22 [26.69%], *NH787* [18.32%]), coefficient of photochemical quenching (qP) (N22 [21.26%], *NH787* [11.26%]), and coefficient of non-photochemical quenching (qN) (N22 [28.66%], *NH787* [15.60%]) (data not shown). Earlier studies also reported the inhibitory effects of Pi deficiency on various photosynthetic and chlorophyll fluorescence traits in the rice genotypes^73,74^, N22 and its EMS mutants^43^. On the contrary, Pi deficiency excreted significant increase in the contents of intercellular CO_2_ (*C*i) (N22 [26.02%], NH787 [26.32%]), chlorophyll a (N22 [35.18%], *NH787* [16.53%]), chlorophyll b (N22 [32.47%], *NH787* [28.70%]), and carotenoid (N22 [18.39%], *NH787* [7.22%]) (data not shown). The result was consistent with an earlier study showing Pi deficiency-mediated elevated content of *C*i^74^. Relatively, the augmenting effects of Pi deficiency on chlorophyll a, b, and carotenoid were significantly lower in *NH787* compared with N22 (data not shown). Under P+ condition, *P*_N_ (27.32%), *g*_s_ (74.66%), *C*i (3.99%), *E* (29.02%), Fv/Fm (3.79%), ETR (15.22%), qN (14.03%), and contents of chlorophyll a (32.74%), chlorophyll b (16.75%), and carotenoid (21.35%) were significantly higher in *NH787* than N22 (Fig. S4A–J). A similar trend was also observed during Pi deficiency where these values (*P*_N_ [19.90%], *g*_s_ [31.97%], *C*i [4.24%], *E* [9.83%], Fv/Fm [12.48%], ETR [28.39%], qP [15.67%], qN [2.46%], and contents of chlorophyll a [14.42%], chlorophyll b [13.43%], and carotenoid [9.91%]) were significantly higher in *NH787* compared with N22 (Fig. 4a–k). The analyses revealed that *NH787* maintained higher photosynthetic and chlorophyll fluorescence traits than N22 under different Pi regimes (Fig. S4, Fig. 4).

### Pi, Apase and the enzymes involved in ROS scavenging of N22 and *NH787* grown to maturity in potting soil

**Figure 5:**
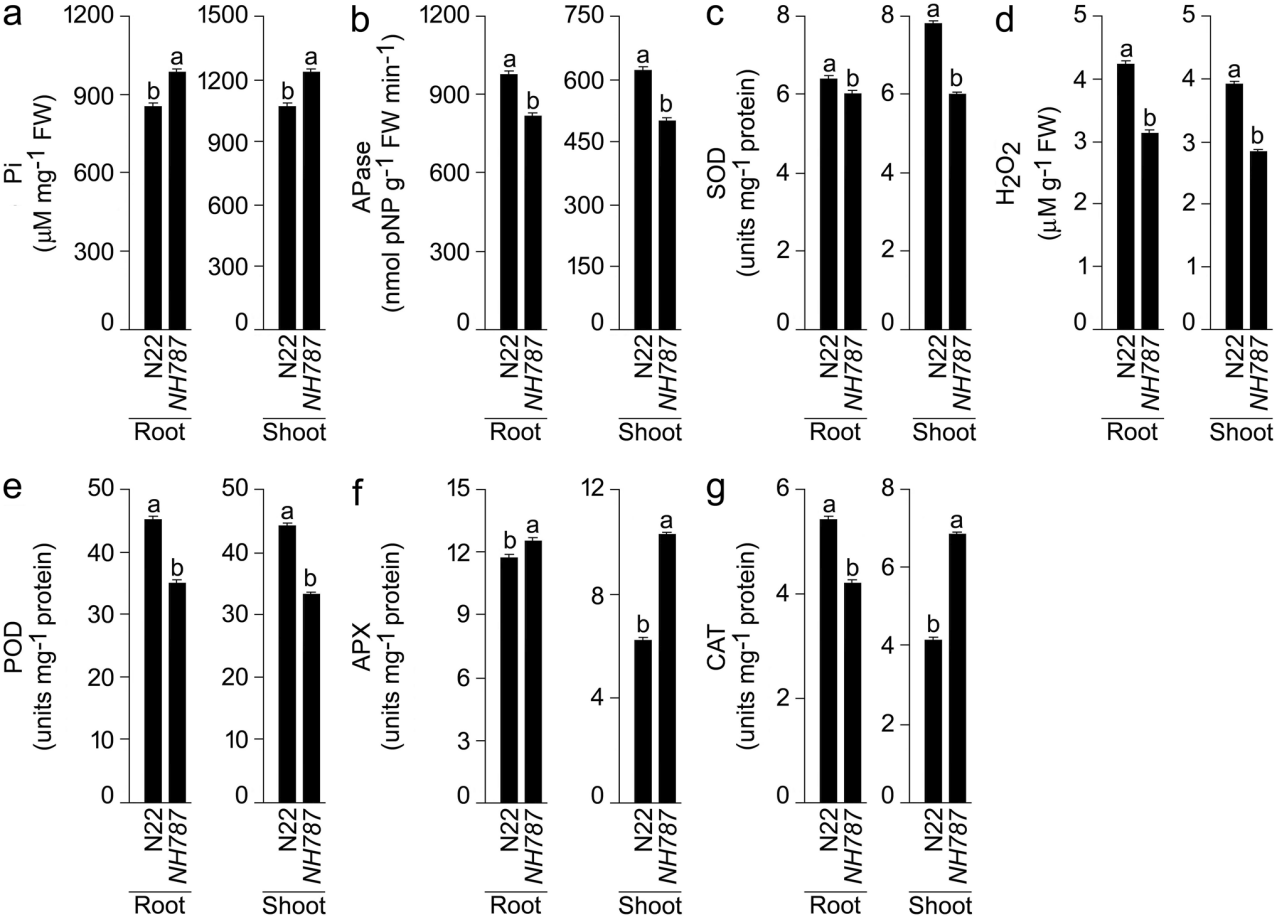
Effects of Pi deficiency on the concentrations of Pi, Apase, and the enzymes involved in ROS scavenging. N22 and *NH787* were grown in a potting soil up to 50% flowering under P+ and P− conditions. (**a**–**g**) Data are presented for the concentrations of (**a**) Pi, (**b**) Apase, (**c**) SOD, (**d**) H_2_O_2_, (**e**) POD, (**f**) APX, and (**g**) CAT. Values (*n* = 12) are means ± SE and different letters on the histograms indicate that the means differ significantly (*P* < 0.05). *Ps* potting soil.

Pi deficiency exerts an attenuating effect on the concentration of Pi, while its effect is augmenting on the activities of Apase and ROS scavenging pathway (APX, CAT, H_2_O_2_, POD, and SOD) in the root and shoot of rice^47^. Therefore, the concentration of Pi and the activities of Apase and ROS scavenging enzymes were assayed in N22 and *NH78*7 grown to maturity under different Pi regimes (Fig. S5, Fig. 5). Pi deficiency triggered significant reductions in the concentration of Pi in the root (N22 [61.30%], *NH787* [55.77%]) and in shoot (N22 [66.86%], *NH787* [65.31%]) (data not shown). The result was consistent with earlier studies reporting Pi deficiency-mediated reduction in the concentration of Pi in the root and shoot of N22 and its EMS mutants^45,47^. It was apparent from this analysis that the effect of Pi deficiency on the concentration of Pi in root was relatively more aggravated in N22 than *NH787* but was comparable in the shoot. Concentration of Pi in the root (P+ [14.90%], P− [31.32%]) and shoot (P+ [15.22%], P− [20.60%]) were significantly higher in *NH787* than N22 (Fig. S5A, Fig. 5a). On the contrary, the activity of Apase increased significantly during Pi deficiency in the root (N22 [2.17 fold], *NH787* [2.86 fold]) and in the shoot (N22 [4.31 fold], *NH787* [4.46 fold]) (data not shown) and was coherent with earlier studies on N22 and its EMS mutants^46,47^. Although the augmenting effect of Pi deficiency on Apase activity was significantly higher in the root of *NH787* compared with N22*,* it was comparable in the shoot of these two genotypes. The activity of Apase in the root (P+ [36.51%], P− [16.28%]) and shoot (P+ [22.17%], P− [19.50%]) were significantly higher in N22 than *NH787* (Fig. S5b, Fig. 5b). Significant augmenting effects of Pi deficiency were also evident in the root and shoot of N22 and *NH787* on different components of ROS pathway comprising SOD (root [41.86% in N22 and 33.59% in *NH787*] and shoot [40.36% in N22 and 50.19% in *NH787*]), H_2_O_2_ (root [2.87 fold in N22 and 2.97 fold in *NH787*] and shoot [69.11% in N22 and 54.03% in *NH787*]), POD (root [40.03% in N22 and 44.97% in *NH78*7] and shoot [82.65% in N22 and 63.84% in NH787]), APX (root [77.63% in N22 and 44.16% in *NH78*7] and shoot [35.90% in N22 and 22.10% in *NH787*]), and CAT (root [74.25% in N22 and 87.84% in *NH78*7] and shoot [2.00 fold in N22 and 2.18 fold in *NH78*7]) (data not shown). The analysis revealed that the values in N22 were significantly higher (root [SOD], shoot [H_2_O_2_ and POD], and root and shoot [APX]) or lower (root [H_2_O_2_ and POD], shoot [SOD], and root and shoot [CAT]) compared with *NH787* (data not shown). Under P+ condition, the values were significantly lower (root and shoot [H_2_O_2_ and POD] and shoot [SOD]), higher (root and shoot [APX and CAT]), and non-significant (root [SOD]) in *NH787* compared with N22 (Fig. S5C–G). Almost a similar trend was observed under P− condition with values significantly lower (root and shoot [SOD, H_2_O_2_, and POD] and root [CAT]) and higher (root and shoot [APX] and shoot [CAT]) in *NH787* compared with N22 (Fig. 5c–g). The results highlighted differential effects on ROS-mediated redox signaling and oxidative stress in *NH787* compared with N22 under different Pi regimes. Earlier studies also showed the Pi-dependent differential effects on ROS homeostasis in the EMS mutants of N22^43,47^.

### Reproductive traits of N22 and *NH787* grown to maturity in potting soil

**Figure 6:**
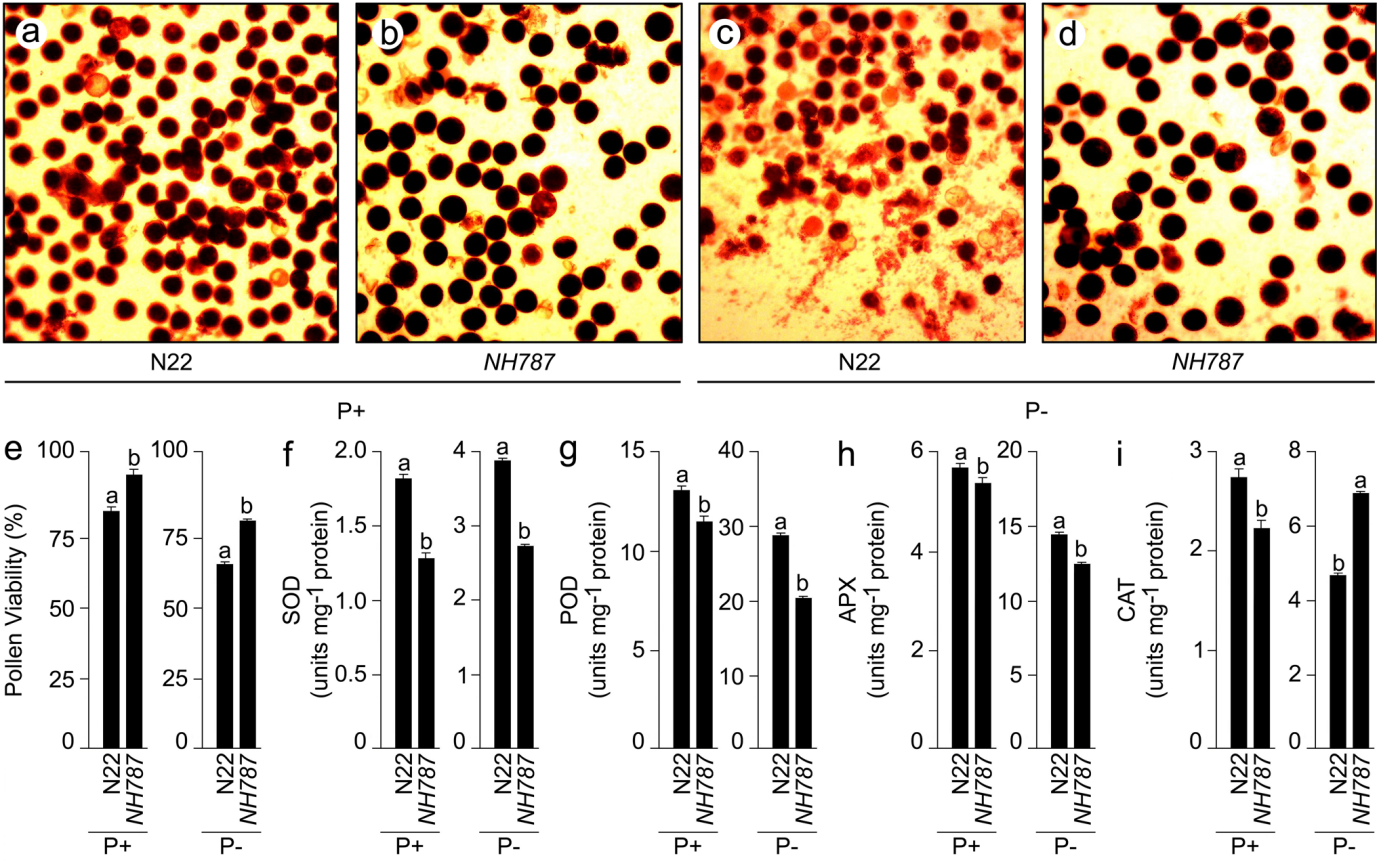
Effects of Pi deficiency on pollen viability and antioxidant enzyme activities in the anther. N22 and *NH787* were grown in a potting soil up to 50% flowering under P+ and P− conditions. (**a**–**d**) Pollen viability was assayed by staining with I_2_-KI and the images were captured with a stereomicroscope. The data are presented for (**e**) Percent pollen viability and (**f**–**i**) the ROS scavenging enzyme activities in the anthers of (**f**) SOD, (**g**) POD, (**h**) APX, and (**i**) CAT. Values (*n* = 6) are means ± SE and different letters on the histograms indicate that the means differ significantly (*P* < 0.05).

**Figure 7:**
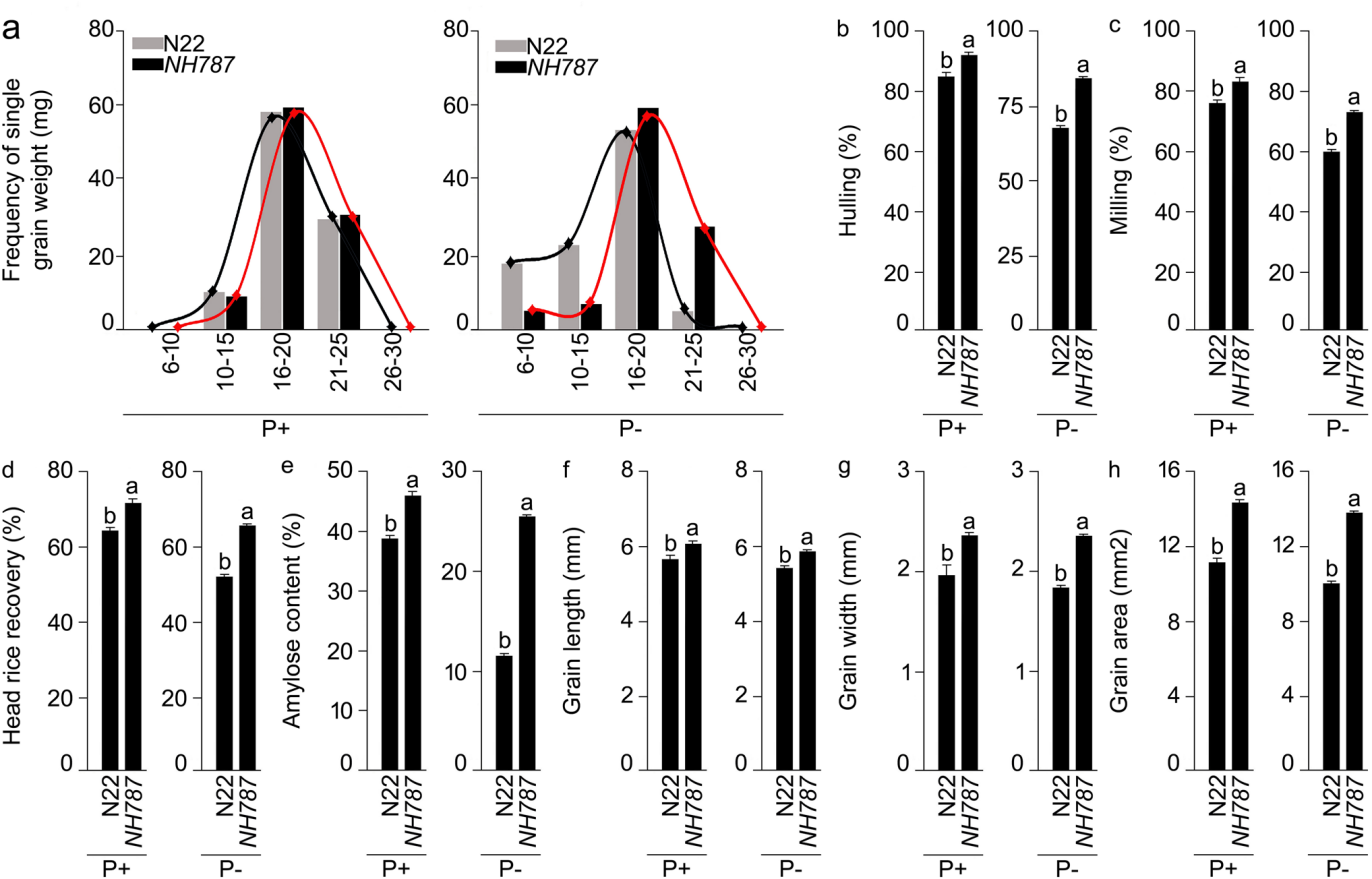
Effects of Pi deficiency on different grain parameters. N22 and *NH787* were grown in a potting soil up to 50% flowering under P+ and P− conditions and after harvesting, grains were threshed, cleaned, and dried under natural conditions. Data are presented for (**a**) frequency of single grain weight, (**b**) percent hulling, (**c**) percent milling (**d**) percent head rice recovery, (**e**) percent amylose content, (**f**) grain length, (**g**) grain width, and (**h**) grain area. Values (*n* = 3, 6, and 20 for (**a**,**b**–**e**), and (**f**–**h**), respectively) are means ± SE and different letters on the histograms indicate that the means differ significantly (*P* < 0.05).

**Figure 8:**
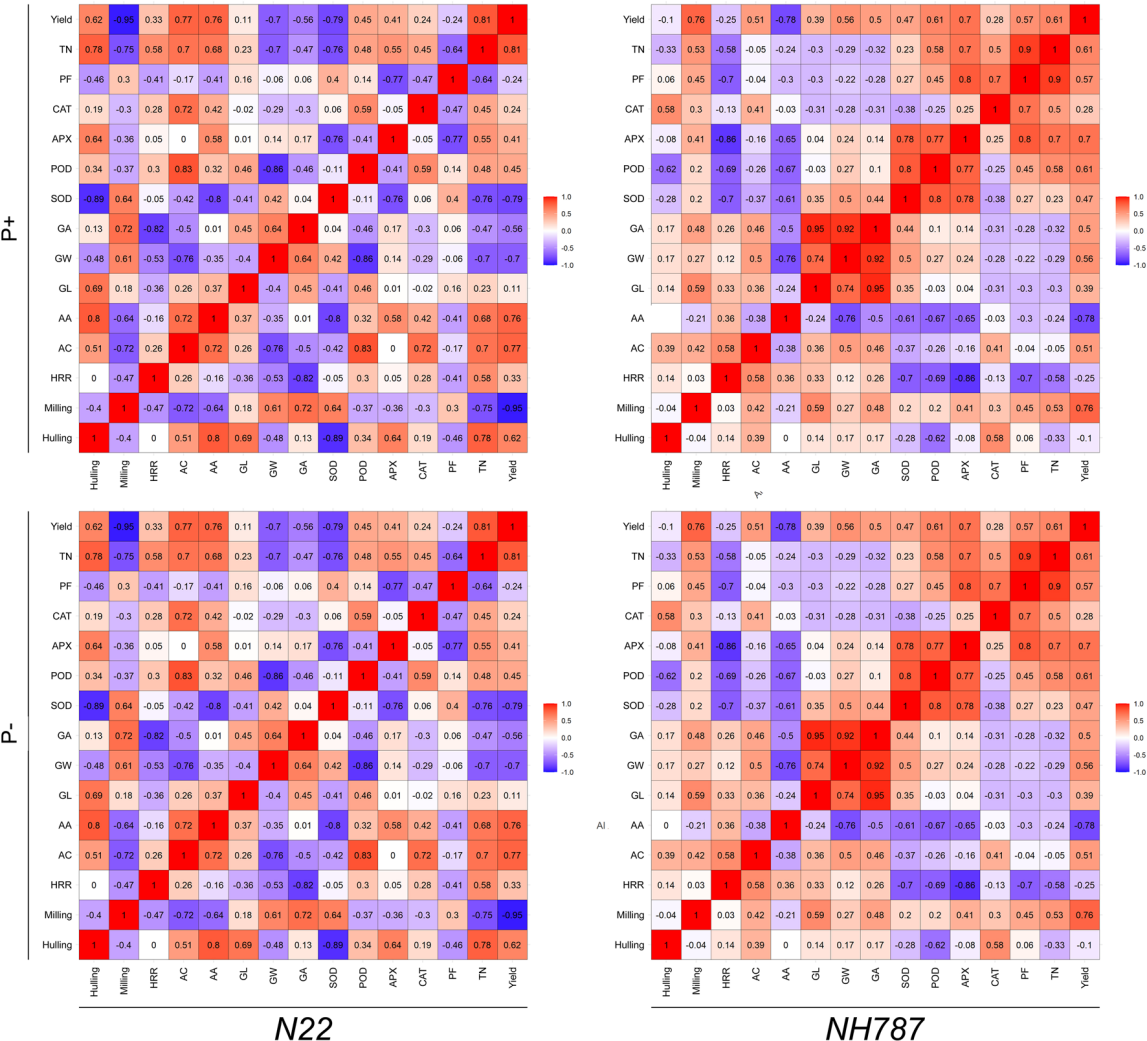
Correlogram of the agronomical and quality traits, pollen fertility, and the activities of antioxidant enzymes and α-amylase in the anther and spikelets, respectively in N22 and *NH787* grown in a potting soil up to 50% flowering under P+ and P− conditions. The scale represents Pearson correlation values with brownish and bluish shades indicate positive and negative correlation, respectively. *TN* Tiller number, *PF* pollen fertility, *GA* grain area, *GW* grain width, *GL* grain length, *AA* α-amylase, *AC* amylose content, *HRR* head rice recovery.

In earlier studies, significant inhibitory effects of Pi deficiency were observed on the yield potential of N22 and its EMS mutants^42–47^. Therefore, the effects of Pi deprivation were investigated on the male reproductive traits of N22 and *NH787* grown under different Pi regimes in a potting soil up to maturity (Fig. 6). I_2_-KI staining was used for determining the viability of pollen collected after anthesis from N22 and *NH787* grown under P+ and P− conditions (Fig. 6a–d). Pollen viability was significantly higher (P+ [14.73%], P− [23.76%]) in *NH787* compared with N22, which suggested that Pi deficiency-mediated effect on pollen viability was more aggravated in the latter than the former (Fig. 6a–E). Further, there were significant reductions in the activities of SOD (P+ [29.48%], P− [29.64%]), POD (P+ [12.58%], P− [29.02%]), and APX (P+ [5.48%], P− [13.63%]) in the anthers of *NH787* compared with N22 irrespective of Pi regimes (Fig. 6f–h). On the contrary, the activity of CAT in *NH787* was significantly lower and higher under P+ (19.05%) and P− (47.05%) conditions, respectively compared with N22 (Fig. 6I). The analysis revealed differential effects on antioxidant enzyme activities of N22 and *NH787* under different Pi regimes. Under Pi-deprived condition, *NH787* exhibited significantly higher CAT activity in the anthers compared with N22, which triggered the efficient conversion of H_2_O_2_ into water and oxygen possibly favoring higher pollen fertility and yield (Fig. S6). Further, the effects of Pi deficiency were determined on various grain parameters of N22 and *NH787* grown in P+ and P− potting soil up to maturity (Fig. 7). Single grain weight of N22 and *NH787* collected from P+ and P− plants were categorized into a week (6–10 mg and 10–15 mg) and robust (16–20 mg, 21–25 mg, and 26–30 mg) categories and the frequency of weight distribution pattern in these categories was computed, which revealed a typical Gaussian curve (Fig. 7a). Under P+ condition, the frequency of single grain weight under different categories was comparable between N22 and *NH787* (Fig. 7a). However, significant variation in the frequency of single grain weight was observed under P− condition for N22 compared with *NH787* ranging from higher (6–10 mg and 10–15 mg), lower (16–20 mg and 21–25 mg), and comparable (26–30 mg) values (Fig. 7a). The analysis revealed that *NH787* seeds were comparatively more robust than N22 when grown under Pi-deprived condition. Under both P+ and P− conditions, several grain quality parameters of *NH787* were significantly higher than N22 comprising hulling (P+ [8.07%], P− [24.18%]) (Fig. 7b), milling (P+ [9.48%], P− [21.21%]) (Fig. 7c), percent head rice recovery (P+ [11.79%], P− [26.54%]) (Fig. 7d), percent amylose content (P+ [18.23%], P− [2.21 fold]) (Fig. 7e), grain length (P+ [7.07%], P− [8.47%]) (Fig. 7f), grain width (P+ [20.30%], P− [27.71%]) (Fig. 7g), and grain area (P+ [28.72%], P− [38.42%]) (Fig. 7h). Different parameters such as alkali spread value, gelatinization temperature, and gel consistency are conventionally used for determining the gain quality traits of rice^75–77^. Therefore, alkali spread value, gelatinization temperature, and gel consistency were assayed for P+ and P− seeds of N22 and *NH787*, which revealed that these traits were superior in the latter compared with the former (Table S2). Although α-amylase activity in the spikelets of N22 and *NH787* was comparable under P+ condition, it was significantly lower in the latter compared with the former under P− condition (Table S3). Low α-amylase activity in the rice spikelets has been correlated with the grain weight and yield^78,79^. Pearson correlation analysis was carried out to determine the relationship across agronomical and quality traits (yield, TN, GA, GW, GL, AC, HRR, milling, and hulling), pollen fertility (PF), and the activities of antioxidant enzymes (CAT, APX, POD, and SOD) and α-amylase (AA) in the anther and spikelets of N22 and *NH787* under different Pi regimes (Fig. 8). Under P+ condition, a positive and significant correlation in N22 was observed with yield, TN, AA, and AC and that of *NH787* with yield, TN, PF, AC, and HRR. Whereas, under P− condition, a positive and significant correlation in N22 was detected with yield, TN, APX, AA, AC, and hulling and that of *NH787* with yield, TN, PF, POD, APX, GA, GW, AC, and milling. The analysis revealed that TN, AC, AA, POD, APX, and PF were correlated positively and significantly with the yield of N22 and *NH787* under different Pi regimes.

### Relative expression levels of GPH in N22 and *NH787* grown to maturity in potting soil

**Figure 9:**
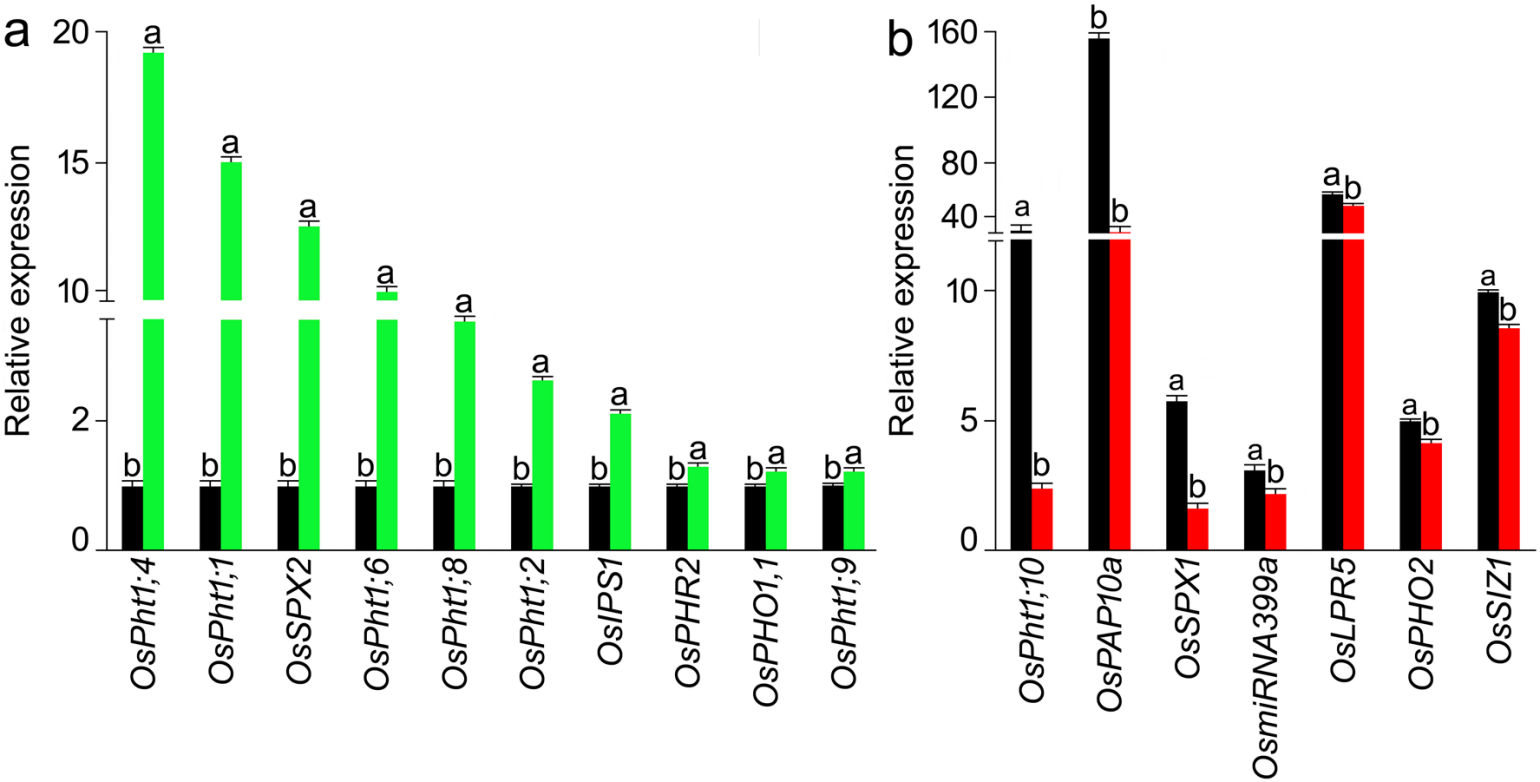
Relative expression levels of GPH in Pi-deprived roots of N22 and *NH787*. N22 and *NH787* were grown in potting soil under P+ and P− conditions up to 50% flowering stage and their roots were harvested. Quantitative real-time RT-PCR (qRT-PCR) was employed for determining the relative expression levels of GPH genes. (**a**) GPH genes induced in *NH787* (green bars) compared with N22 (black bars). (**b**) GPH genes were suppressed in *NH787* (red bars) compared with N22 (black bars). *OsActin* (LOC_Os03g50885) was used as an internal control. Values are means ± SE (*n* = 6) and different letters on the histograms indicate that the values differ significantly (*P* < 0.05).

The qRT-PCR assay was employed to decipher Pi deficiency-mediated effects on the relative expression levels of functionally diverse GPH in the roots of N22 and *NH787* grown to maturity in potting soil under P+ and P− conditions (Fig. 9). For this experiment, only those GPH were selected, which had been functionally characterized either by overexpression under the constitutive promoter or by mutation (T-DNA, *Tos17*, RNAi, or CRISPR-cas9) and implicated in their tissue-specific key roles in the sensing and signaling cascades governing the maintenance of Pi homeostasis under Pi regimes (Table 2). In Pi-deprived roots of *NH787* compared with N22, the relative expression levels of several GPH were significantly higher that are implicated in the transcriptional regulation of signaling pathway (*OsPHR2*^80^), regulation by systemic and local Pi signaling and hormones (*OsIPS*^81^), regulation of Fe transport by integrating Pi and Zn deficiency signaling (*OsPHO1;1*^82^), inhibition of Pi starvation responses by interacting with *OsPHR2* in a Pi-dependent manner (*OsSPX2*^83^), and uptake and/or mobilization of Pi by low-and high-affinity Pi transporters (*OsPht1;1*, *OsPht1;2*, *OsPht1;4*, *OsPht1;6*, *OsPht1;8*, and *OsPht1;9*^50,84–89^ (Fig. 9a). Higher relative expression levels of these GPHs in *NH787* could be assumed to play a pivotal role in contributing towards its higher yield potential (Fig. 3). On the contrary*,* the relative expression levels of several GPH were significantly reduced in Pi-deprived roots of *NH787* compared with N22 that are involved in the uptake of Pi by high-affinity Pi transporter (*OsPht1;10*^87^), utilization of extracellular organic P (*OsPAP10a*^90^), regulation of the growth during Pi deficiency via a negative feedback loop and by interacting with *OsPHR2* in a Pi-dependent manner (*OsSPX1*^83,91^), regulation of Pi starvation signal transduction (*OsmiRNA399a*^92^), growth, development, and maintenance of Pi homeostasis (*OsLPR5*^93^), regulation of Pi starvation responses (*OsPHO2*^94^), and post-translational SUMOylation of proteins (*OsSIZ1*^95^) (Fig. 9b). In *Arabidopsis thaliana*, electrophoretic mobility shift assay revealed the binding of the transcription factor PHR1 as a dimer to an imperfect palindromic 8-bp sequence (5′-GNATATNC-3′) named as PHR1 binding sequence (P1BS) found in the promoter (2 kb upstream of ATG start codon) of several genes involved in Pi deficiency-mediated responses^96,97^. Therefore, the P1BS (GNATATNC) motif was analyzed in the promoter (3 kb upstream of ATG initiation site) of the 17 GPH revealing its presence in 14 of them, which suggested their potential regulation by OsPHR2 (Table 2). In this context, significantly higher relative expression of *OsPHR2* in Pi-deprived roots of *NH787* compared with N22 (Fig. 9a) suggested its potential regulatory influence on the expression of several GPH that play a key role in the maintenance of Pi homeostasis under different Pi regimes.

## Conclusions

**Figure 10:**
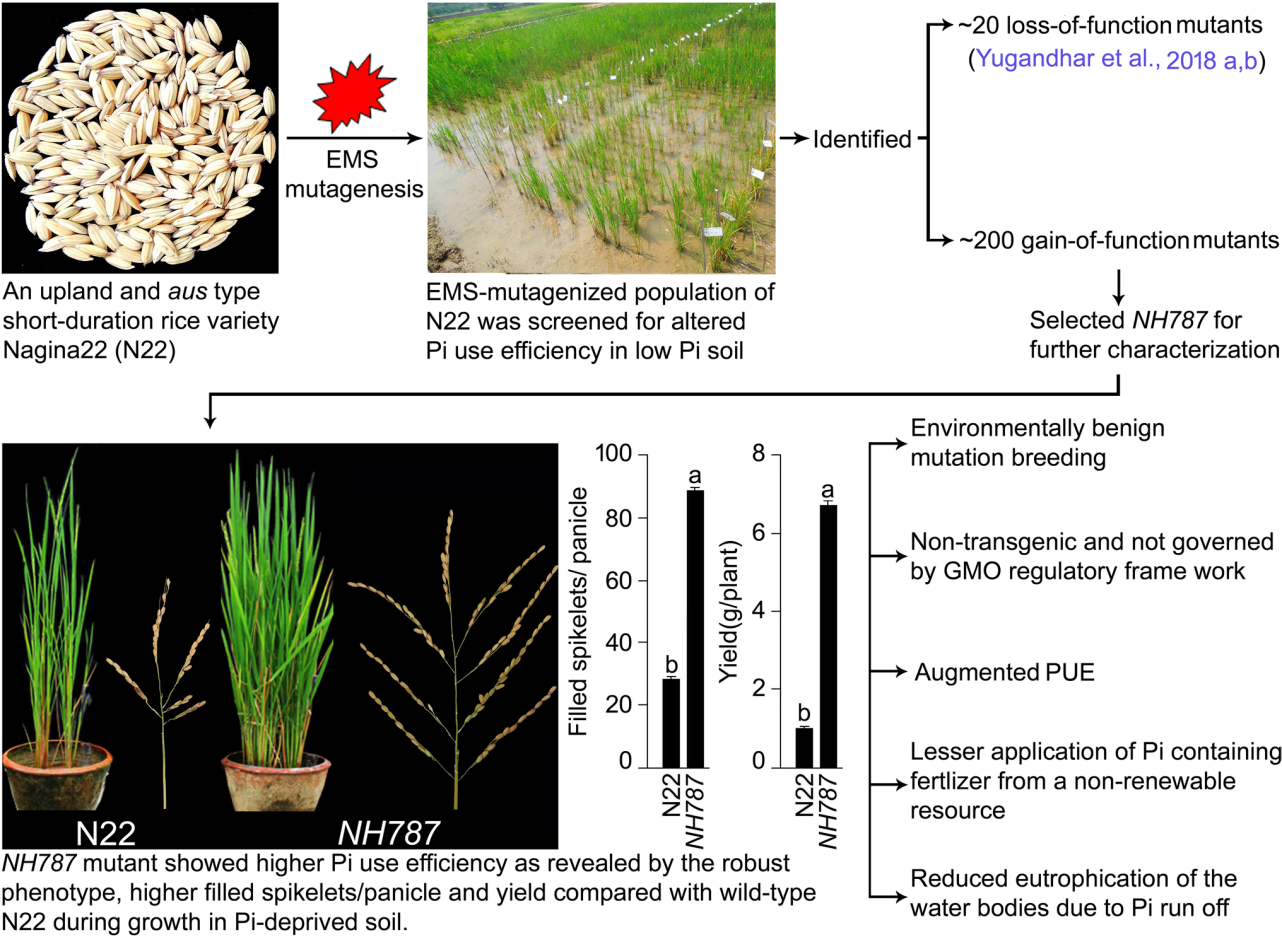
A schematic representation highlighting the efficacy of the EMS mutants for screening the rice variety *NH787* with higher PUE in an environment-friendly manner for sustainable production.

The results provided empirical evidence towards the differential effects of the EMS mutagenesis on various morphophysiological, biochemical, and molecular traits of *NH787* that conferred higher PUE under low Pi soil condition compared with N22. A schematic diagram is presented highlighting the efficacy of the EMS mutants for screening the rice variety *NH787* with higher PUE in an environment-friendly manner for sustainable production (Fig. 10). *NH787* is now used as a donor in breeding programs for developing low P tolerant varieties with superior grain quality and is also being evaluated in larger plots at multiple locations with variable agroclimatic conditions. Efforts are also underway to identify the candidate genes in *NH787* responsible for higher PUE by employing quantitative trait loci (QTL) mapping and MutMap approach in the F2 populations revealing a discernible phenotype^98^. MutMap approach has been used in an earlier study for identifying the candidate genes conferring salt tolerance in the F2 populations of EMS mutant *hitomebore salt tolerant 1* (*hst1*) of rice variety Hitomebore^99^.

## Supplementary Information

Supplementary Information.

## Data Availability

All data generated or analyzed during this study are included in this published article (and its Supplementary Information files). The sequence data is available on request.
