# Supplementary material for: NH787 EMS mutant of rice variety Nagina22 exhibits higher phosphate use efficiency
Source: Sci Rep. 2021 Apr 28;11:9156. doi: 10.1038/s41598-021-88419-w (PMC8080636; doi:10.1038/s41598-021-88419-w)
Supplement: Supplementary file 1 — Supplementary Information. [file 41598_2021_88419_MOESM1_ESM.docx]

*NH787* EMS mutant of rice variety Nagina22 exhibits higher phosphate use efficiency

Poli Yugandhar^1^, Nallamothu Veronica^1^, Hao Ai^2^, Muddapuram Deeksha Goud^3^, Xiaowen Wang^2^, Desiraju Subrahmanyam^1^, Mangrauthia Satendra K^1,**^ & Jain Ajay ^3,*^

^1^ ICAR-Indian Institute of Rice Research, Hyderabad, 500030, India. ^2^State Key Laboratory of Crop Genetics and Germplasm Enhancement, Key Laboratory of Plant Nutrition and Fertilization in Low-Middle Reaches of the Yangtze River, Ministry of Agriculture, Nanjing Agricultural University, 210095, Nanjing, China. ^3^Amity Institute of Biotechnology, Amity University Rajasthan, Jaipur, India.Correspondence and requests for materials should be addressed to A.J. (email: ajain2@jpr.amity.edu)

⁎ Corresponding author, ** Co-corresponding author


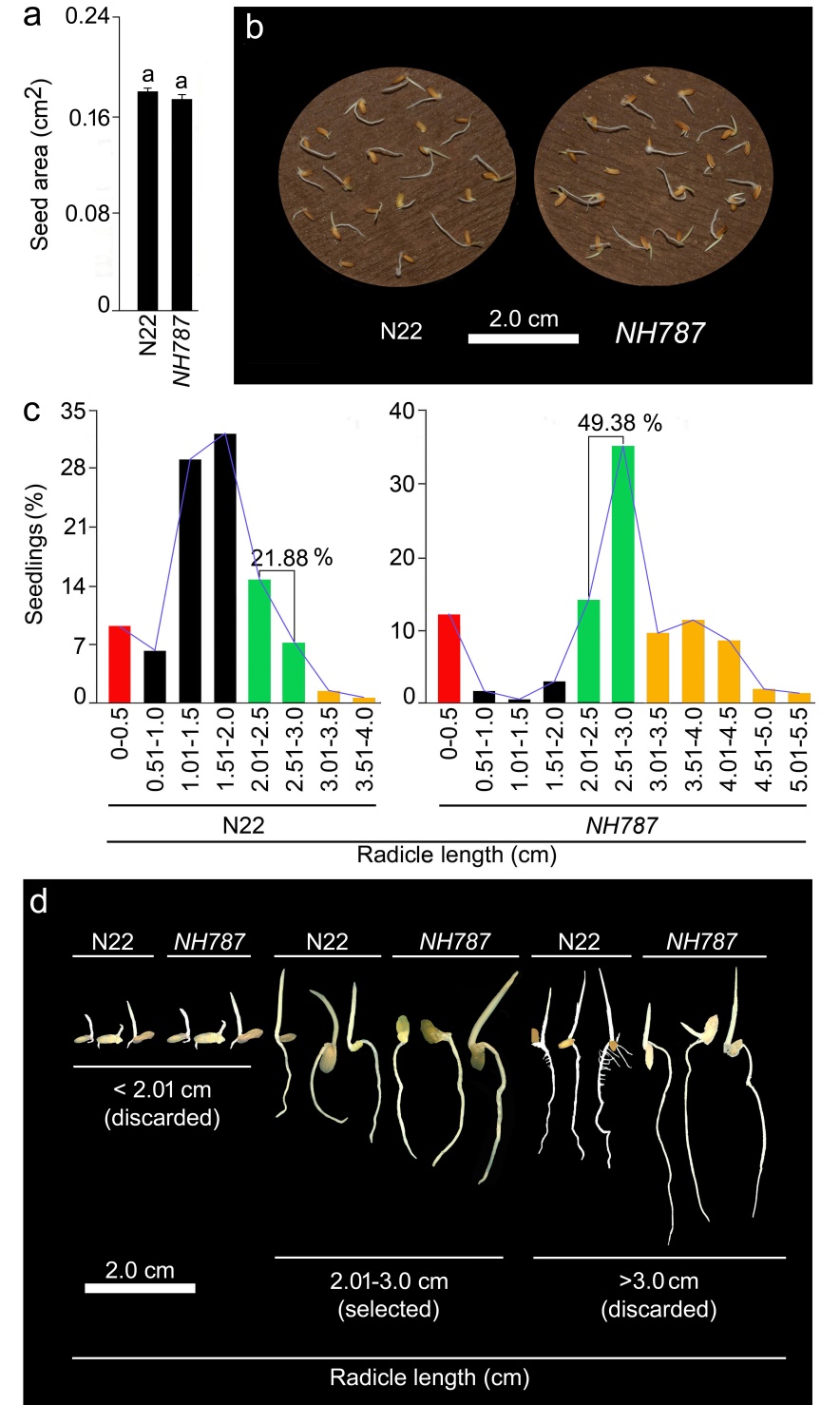


**Figure S1.** Selection of N22 and *NH787* seedlings with minimum variability in their radical length. (a) Data presented for seed area. Values are means ± SE (*n* = 200) and different letters on the histograms indicate that the values differ significantly (*P*< 0.05). (b) Germinated seedlings (4-d-old) in a Petri plate lined with moist germination towel. (c) Different size ranges of the radicle length of the germinated seedlings revealing a typical Gaussian curve distribution. Red, black, green, and yellow histograms represented the seedlings with radicle length ≤ 0.5 cm, 0.51-2.0 cm, 2.01-3.00 cm, and 3.01-5.5 cm, respectively. The values on the top of the green histogram indicated the per cent seedlings that were selected for the subsequent treatments. The rest of the seedlings represented by red, black, and yellow histograms were discarded. (d) The phenotype of the seedlings selected for the treatments under P+ and P- conditions (middle section) and those discarded (flanking sections).

**
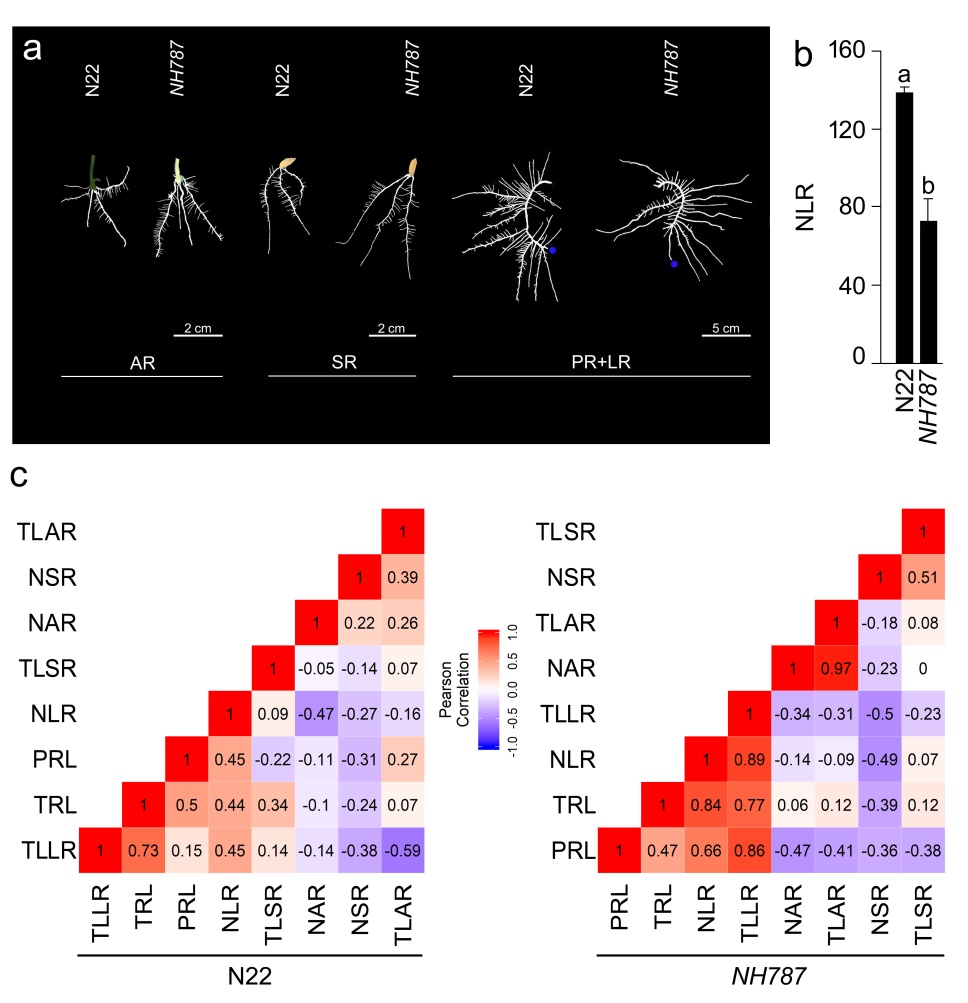
**

**Figure S2.** RSA traitsof N22 and *NH787* seedlings under P+ condition. (a) N22 and *NH787* seedlings (4-d-old) were grown hydroponically under P+ condition. Roots were spread gently to reveal the architectural details of the adventitious (AR), seminal (SR), primary (PR), and lateral (LR) roots. The primary root tip is indicated by a blue dot. (b) Data presented for the number of lateral roots (NRL). Values (*n* = 12) are means ± SE and different letters on the histograms indicate that the means differ significantly (*P*< 0.05). (c) Correlogram of the RSA traits under P+ condition. The scale represents Pearson correlation values with bluish and reddish shades indicate positive and negative correlation, respectively.


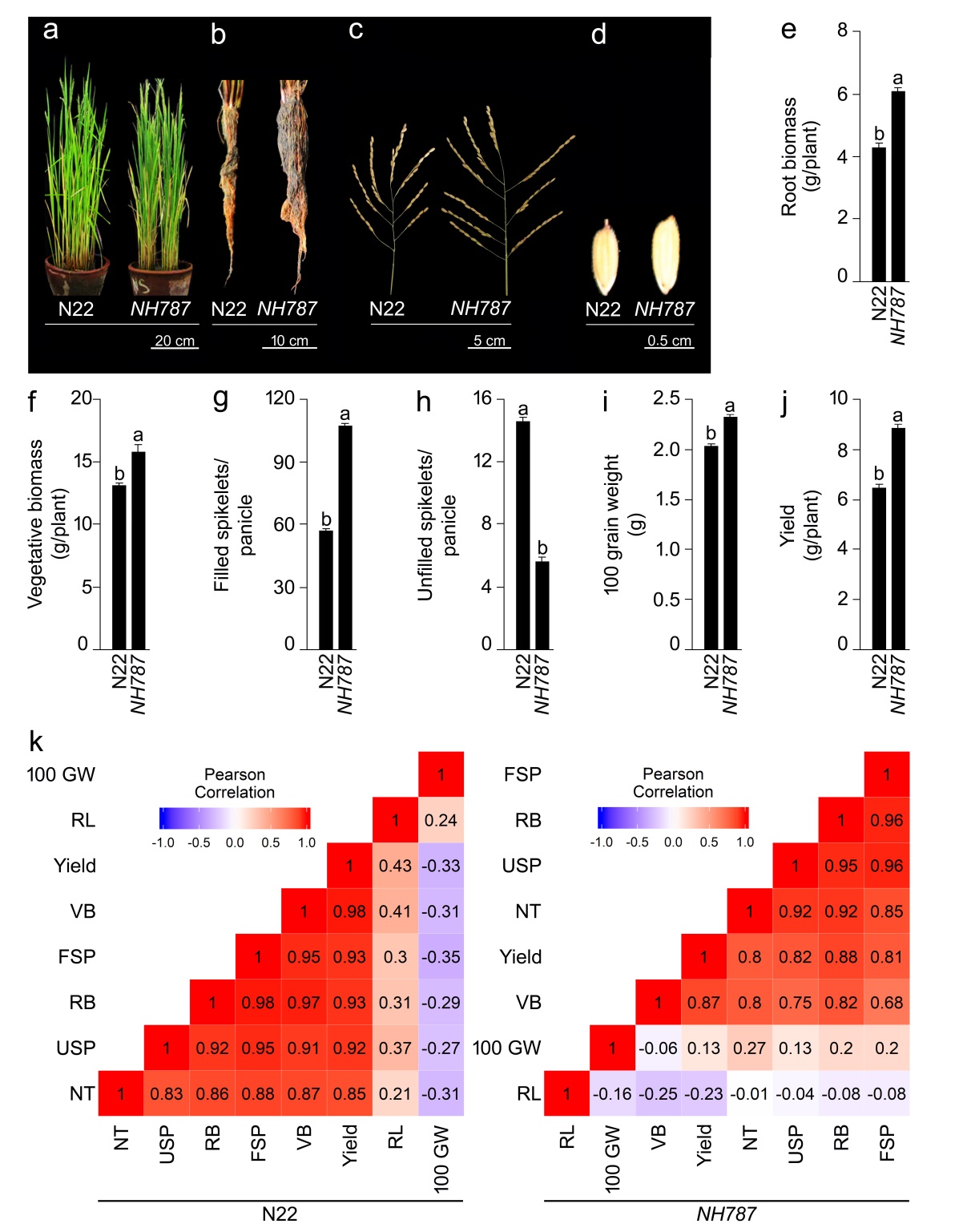


**Figure S3.** Growth performance and the agronomic traits of N22 and *NH787* grown to maturity in Pi-replete potting soil. (a-d) Growth performance of the mature plants (a), and the phenotype of the root (b), panicle (c), and seed (d)*.*The photographs (a-d) are representatives of 12 independent biological replicates. (e-j) Data presented for (e) Root biomass, (f) Vegetative biomass, (g) Filled spikelets/panicle, (h) Unfilledspikelets/panicle, (i) 100 grain weight, and (j) Yield. Values (*n* = 12) are means ± SE and different letters on the histograms indicate that the means differ significantly (*P* < 0.05). Correlogram of the agronomic traits i.e., 100-grain weight (100 GW), Root length (RL), Yield, Vegetative biomass (VB), Filled spikelets/panicle (FSP), Root biomass (RB), Unfilled spikelets/panicle (USP), and the number of tillers (NT) in Pi-sufficient N22 and *NH787.* The scale represents Pearson correlation values with bluish and reddish shades indicate positive and negative correlation, respectively.


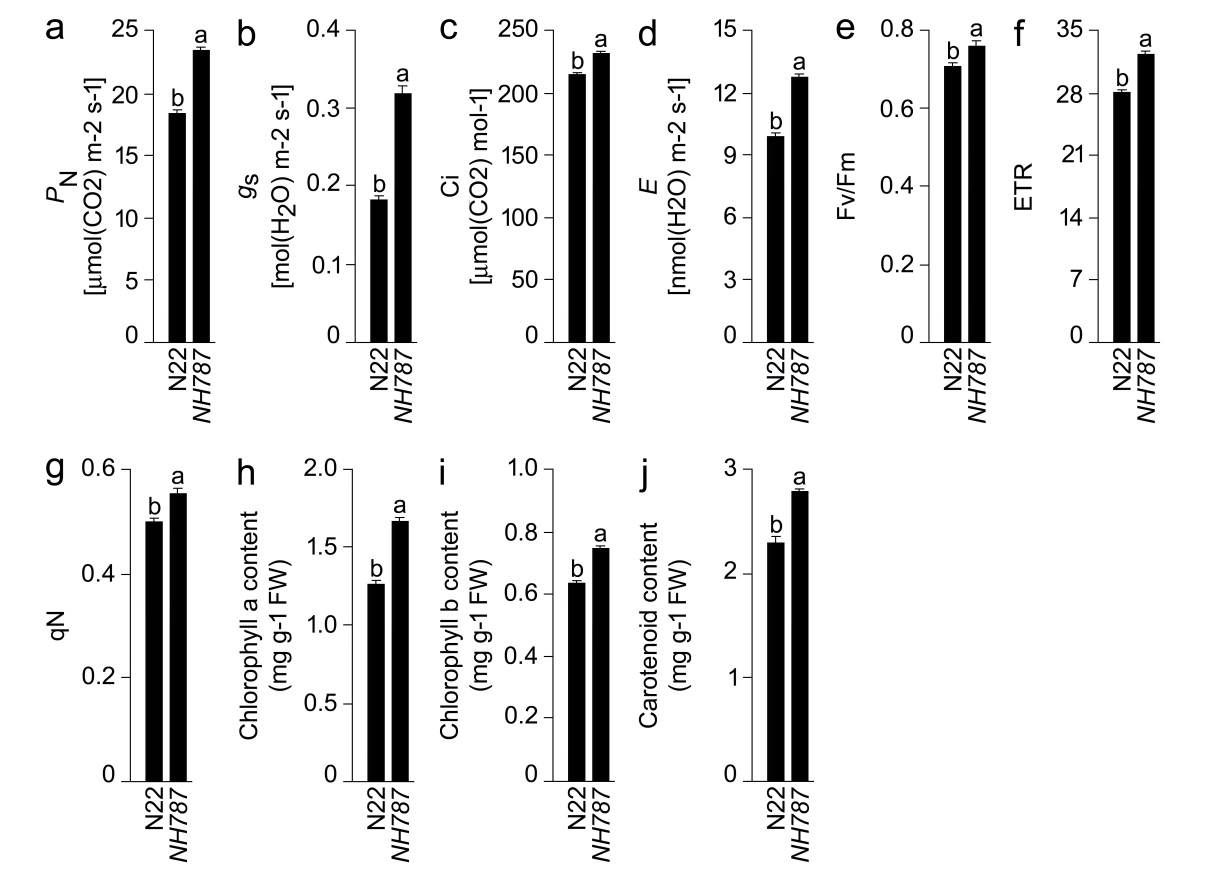


**Figure S4.** Photosynthetic and fluorescence traits of N22 and *NH787* grown to maturity in Pi-replete pot soil. (a-j) Data are presented for (a) Photosynthetic rate (*P*_N_), (b) Stomatal conductance (*g*_s_), (c) Intercellular CO_2_ concentration (Ci), (d) Transpiration rate (*E*), (e) Maximum efficiency of PSII photochemistry (Fv/Fm), (f) Electron transport rate (ETR), (g) Coefficient of non-photochemical quenching (qN), and the contents of (h) Chlorophyll a, (i) Chlorophyll b, and (j) Carotenoid. Values (*n* = 12) are means ± SE and different letters on the histograms indicate that the means differ significantly (*P* < 0.05).


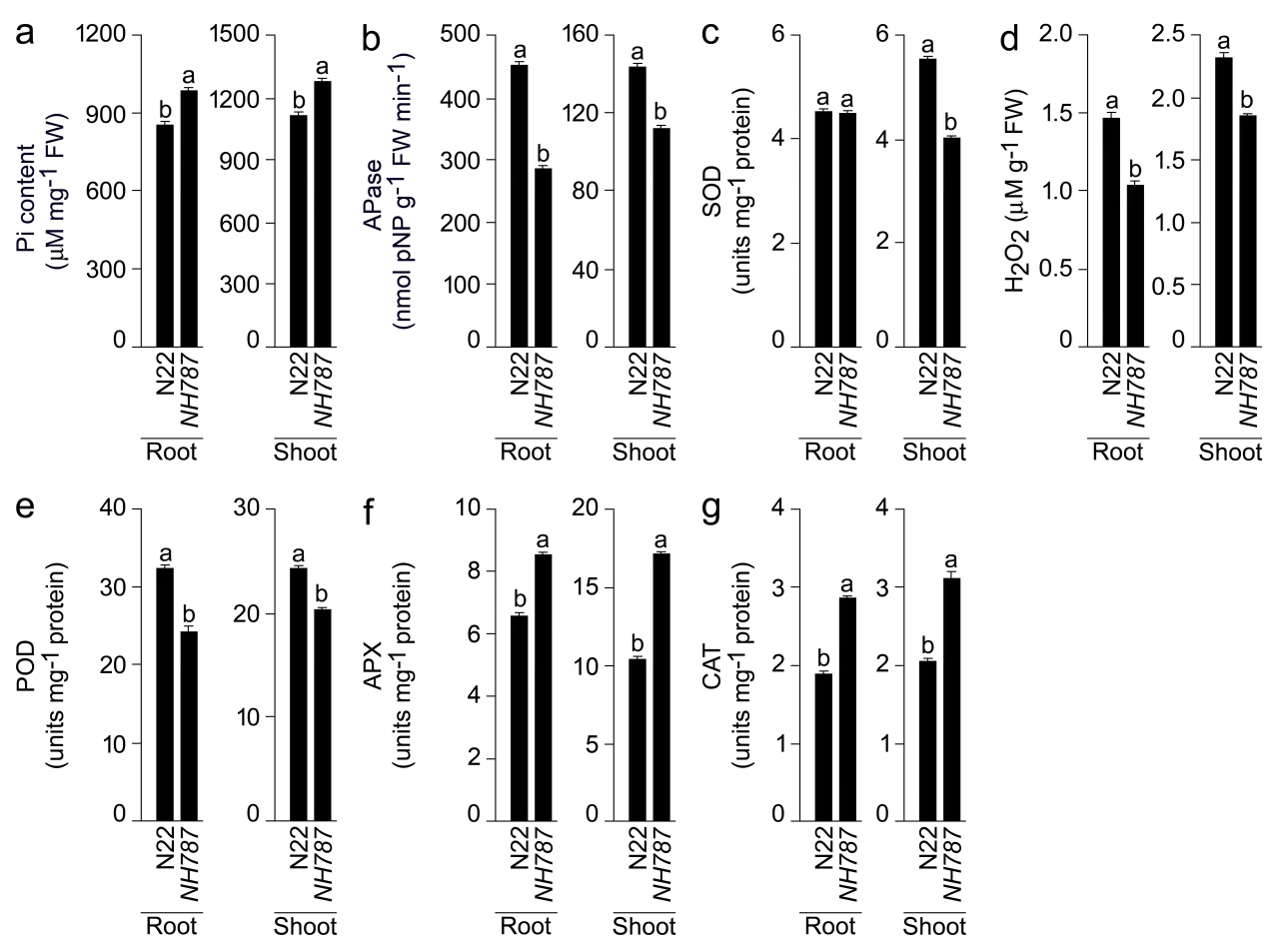


**Figure S5.** Concentrations of Pi, Apase, and the enzymes involved in ROS scavenging in N22 and *NH787*grown to maturity in Pi-replete pot soil. (a-g) Data are presented for the concentrations of (a) Pi, (b) Apase, (c) SOD, (d) H_2_O_2_, (e) POD, (f) APX, and (g) CAT. Values (*n*=12) are means ± SE and different letters on the histograms indicate that the means differ significantly (*P* < 0.05).


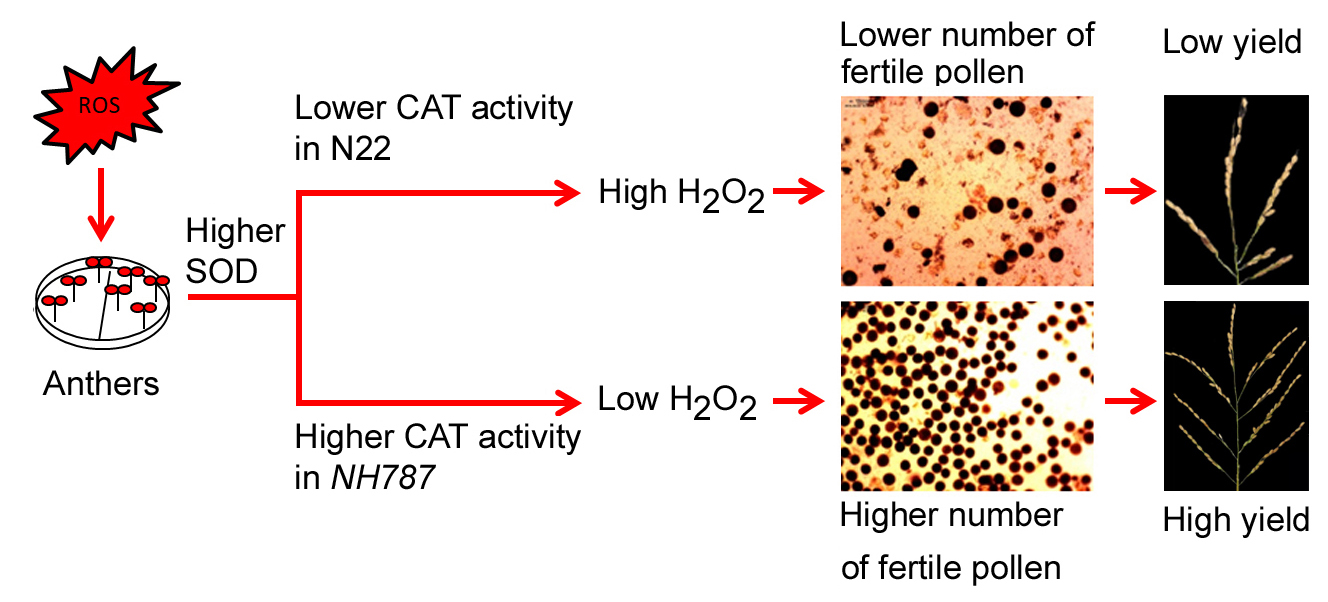


**Figure S6.** Schematic diagram showing the effect of higher and lower CAT activities in the anthers of N22 and *NH787* on the pollen fertility and yield.

**Supplementary Table 1.** The primers used for qRT-PCR.

| Genes | Primer sequence (FP) | Primer sequence (RP) |
| --- | --- | --- |
| *OsPht1,4* | TTCTGCTAGTGTACCAAACAAAATTACA | GTAAGTGGCATTTATAATATCAACAGTAACC |
| *OsPht1,1* | CAGTGCTGGATGGCTGTCACTT | GCTTCCGATGTTAGGGTGGATG |
| *OsSPX2* | GGAGGTGAAAACGAGAATGG | ACAGCAGGTGGGAAACAAAC |
| *OsPht1,6* | CGCTTCCGTACGAGTGGTAGT | GGTTCTTTCAAATCCAGGGAAA |
| *OsPht1,8* | GACGAGACCGCCCAAGAAG | TTTTCAGTCACTCACGTCGAGAC |
| *OsPht1,2* | CAACACCCCTGCTATGTACG | CATCACCAGAGTCCAACACAA |
| *OsIPS1* | CGCCTAGCATGGACTGAGAGTGT | GTTCACGGAATGGTAATGGGACA |
| *OsPHO1,1* | AAGACGGTGGTTCGATGAGAGC | TGATCCAAGGCTCCTGTCCTTC |
| *OsPht1,9* | AGAAAAACATAGGCTTGTCATCCTTT | AAAACCTAAGAAGCACTGTAAATAAATCC |
| *OsPht1,10* | ATGTCGCCCATCCTTCCA | TCGCTTTCCGACGATGATC |
| *OsPAP10a* | ATACTGGCAGCCGACGGATGA | GAGGGAGCTGGAGCGGAGAA |
| *OsSPX1* | CGACTTCCATGGCGAGAT | TCCTCTTGTCATACTTCTTGAGAATC |
| *OsPHR2* | TGCGACTGCTGTATTCAGTACGT | ACAAATGCCATCAAATATGAACAGA |
| *MiRNA399a* | ACAGCAGGTGGGAAACAAAC | GCTGGAAATGATGCTGGTAGC |
| *OsLPR5* | CGATGAGAATATGAGATGAAGAAGCT | CGCACCAGTTTATGACTAGCAAA |
| *OsPHO2* | GGCTATCGGAACTTATGG | AAGAAGGCAGAGGAGGTATC |
| *OsSIZ1* | AAAGCTGCCATCCAGAAATCAT | AGCTTGTAAACCATTGAGGCAAC |
| *OsActin* | AAGACGGTGGTTCGATGAGAGC | TGATCCAAGGCTCCTGTCCTCC |

**Supplementary Table 2.** Alkali spread value, gelatinization temperature, and gel consistency of N22 and *NH787* grown under P+ and P- conditions. Values (*n* = 6) are means and different superscript letters indicate that the means differ significantly (*P* < 0.05).

|  | Alkali spread value | | Gelatinization temperature | | Gel consistency | |
| --- | --- | --- | --- | --- | --- | --- |
|  | P+ | P- | P+ | P- | P+ | P- |
| N22 | Soft | Soft | Soft | Soft | Soft | Soft |
| *NH787* | Intermediate | Intermediate | Intermediate | Intermediate | Intermediate | Intermediate |

**Supplementary Table 3.** Activity of α-amylase in N22 and *NH787* grown under P+ and P- conditions. Values (*n* = 6) are means and different superscript letters indicate that the means differ significantly (*P* < 0.05).

|  | α-amylase activity  (U g/protein) | |
| --- | --- | --- |
|  | P+ | P- |
| N22 | 9.41^c^ | 15.15^a^ |
| *NH787* | 9.29^c^ | 13.44^b^ |
